# Overcoming Interfacial Hurdles in Solid‐State Aluminum Batteries for Safe and Energy‐Dense Storage

**DOI:** 10.1002/advs.76667

**Published:** 2026-07-17

**Authors:** Yunlei Wang, Xiaogang Zhang, Mingguang Wang, Yifan Wu, Shitao Dou, Taibin Wu

**Affiliations:** ^1^ School of Materials Science and Engineering Chongqing University of Arts and Sciences Chongqing China; ^2^ Department of Mechanical and Materials Engineering University of Western Ontario London Ontario Canada; ^3^ Institute for Advanced Materials and Technology University of Science and Technology Beijing Beijing China

**Keywords:** energy density, interface design, solid‐state aluminum batteries (SSABs), solid‐state electrolytes

## Abstract

Solid‐state aluminum batteries (SSABs), leveraging the high theoretical capacity, natural abundance, and intrinsic safety of the metallic aluminum anode, are regarded as a highly promising energy storage system for the post‐lithium era. However, the severe solid–solid interface issues between solid‐state electrolytes and electrodes, including high interfacial impedance, sluggish ion transport kinetics, and uncontrollable aluminum dendrite growth, significantly constrain their practical energy density and cycling stability, representing a critical bottleneck toward commercialization. This review aims to systematically elucidate the physicochemical origins of the multi‐scale interfacial challenges in SSABs, with a focused discussion on the latest breakthrough strategies in interface design and engineering. It provides an in‐depth analysis of how constructing artificial interphases, designing gradient composite electrolytes, tailoring interfacial ion transport pathways, and introducing advanced characterization techniques can effectively promote uniform aluminum plating/stripping, suppress side reactions, and achieve stable interfacial contact. Furthermore, this work prospectively discusses feasible pathways toward compatible high ionic conductivity, excellent mechanical strength, and robust electrochemical stability through integrated interface architectures and synergistic electrode/electrolyte design. Finally, we distill the key challenges and future opportunities spanning from fundamental understanding to device integration, aiming to provide a clear roadmap for developing next‐generation SSABs that combine high‐energy density with long‐term operational safety.

## Graphical abstract

Solid‐state aluminum batteries (SSABs) promise high energy density and intrinsic safety but face severe solid–solid interfacial challenges. This review reveals the three core hurdles—anode passivation, cathode contact loss, and electrolyte grain boundary resistance and discusses strategies including artificial interphases, alloying, and composite electrolyte design. These approaches collectively enable uniform aluminum deposition, suppress side reactions, and improve cycling stability, paving the way toward practical high‐performance devices.

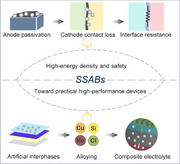

## 1 Introduction

The green transformation of the global energy structure is advancing at an unprecedented pace. The large‐scale deployment of renewable energy and the booming development of the electric vehicle industry are placing higher demands on electrical energy storage technologies: higher energy density, lower cost, improved safety, and more sustainable material systems [1, 2, 3]. The currently dominant lithium‐ion batteries (LIBs) are gradually approaching their theoretical energy density limits while simultaneously facing supply chain challenges such as uneven global distribution and volatile prices of critical resources like lithium and cobalt [4, 5]. Against this backdrop, the exploration of the post‐lithium battery era has become a strategic focal point in global energy materials research.

Among numerous candidate technologies, multivalent ion batteries have attracted significant attention due to their characteristic of each ion transferring multiple electrons, theoretically offering higher volumetric energy density and lower material costs [6]. Among multivalent metal anodes such as magnesium, zinc, calcium, and aluminum [7, 8, 9, 10], the aluminum metal anode exhibits particularly outstanding comprehensive advantages: First, aluminum features a three‐electron transfer mechanism, with a theoretical specific capacity as high as 2980 mAh/g (approximately four times that of lithium) and a volumetric capacity of 8046 mAh/cm^3^ (approximately five times that of lithium) [11]. Second, aluminum is the most abundant metal element in the Earth's crust (∼ 8.2%), offering low cost and a mature supply chain [12]. More importantly, aluminum metal is stable in air and less prone to violent reactions with common electrolytes, fundamentally avoiding safety hazards associated with lithium metal [13], such as volatility and combustion, and the comprehensive comparison of typical characteristics for multivalent metal battery systems (Mg, Zn, Ca) vs. SSABs is shown in Table 1. These characteristics position aluminum batteries as one of the ideal candidates for realizing low‐cost, high‐safety, high‐energy‐density energy storage systems [14, 15].

**TABLE 1:** Comprehensive comparison of typical characteristics for multivalent metal battery systems (Mg, Zn, Ca) vs. SSABs.

Feature / Parameter	Mg‐ion	Zn‐ion	Ca‐ion	SSABs
Ionic radius (Å)	∼ 0.72	∼ 0.60	∼ 1.00	∼ 0.54 (Smaller)
Theoretical Volumetric Capacity (mAh cm^−3^)	3832	5855	2073	∼ 8040 (Highest)
Theoretical Gravimetric Capacity (mAh g^−1^)	2205	820	1337	2980
Earth Crust Abundance (Rank)	6th	24th	5th	3rd (Most abundant)
Standard Redox Potential (vs. SHE)	−2.37 V	−0.76 V	−2.87 V	−1.66 V (Moderate, safer)
Major Challenges	Severe passivation, immobile Mg^2+^ in lattices	Dendrite growth, narrow electrochemical window	Highly reactive with moisture/electrolytes	AlCl_4_^−^ / Al^3+^ complex chemistry (Highlight this is solved by your solid‐state design)
Safety and Stability	Moderate (Flammable organics)	Moderate (HER issues)	Low (Air‐sensitive)	High (Non‐flammable SSB)

However, conventional aluminum‐ion batteries (AIBs) employing liquid electrolytes face severe challenges: the aluminum anode surface is prone to passivation from ambient exposure (native Al_2_O_3_) and/or from reactions with solid‐state electrolytes [16], leading to high overpotential and low coulombic efficiency; the solid‐state diffusion of aluminum ions within cathode materials is slow, limiting overall kinetic performance [17, 18]. Furthermore, the growth of aluminum dendrites and side reactions with the electrolyte further constrain cycle life. Among various electrolyte systems for conventional AIBs, chloroaluminate ionic liquids (ILs), particularly AlCl_3_/EMIC (1‐ethyl‐3‐methylimidazolium chloride), remain the most widely studied due to their ability to reversibly electrodeposit aluminum at room temperature. However, these ILs suffer from a series of inherent defects that severely impede their practical application and large‐scale commercialization. These issues have prompted researchers to turn their attention to a more revolutionary system—Solid‐state aluminum batteries (SSABs) [19, 20]. Additionally, the classification and advantages of SSABs into three types based on electrolyte composition: polymer‐based, inorganic (ceramic)‐based, and composite/hybrid electrolytes. For each category, it clearly articulates their respective advantages, particularly intrinsic safety (elimination of leakage, combustion, and explosion risks), compatibility with high‐voltage cathodes, and the potential to suppress dendrite penetration, which fundamentally distinguishes SSABs from their ionic liquid‐based counterparts.

The vision of SSABs carries a dual promise for energy storage technology. On one hand, by replacing flammable liquid electrolytes with solid‐state electrolytes [21], intrinsic safety can potentially be achieved, completely eliminating risks of leakage, combustion, and explosion [22]. On the other hand, the solid‐state system enables the direct use of high‐capacity aluminum metal anodes [23], thereby unlocking their full theoretical energy density potential, with the prospect of breaking the 500 Wh/kg energy density barrier to meet the stringent requirements for long‐range electric vehicles and long‐duration grid energy storage [24].

Behind this grand promise lie the inherent theoretical advantages of solid‐state batteries [25, 26], a wide electrochemical window allows for matching high‐voltage cathodes, the solid medium can suppress dendrite penetration growth, and the absence of fluidity eliminates the continuous occurrence of interfacial side reactions. However, as we shift our focus from the theoretical blueprint to the laboratory bench, a vast chasm becomes apparent, SSABs currently remain largely in the laboratory concept stage, with their actual performance far below theoretical expectations. Key metrics such as cycle life, rate capability, and energy efficiency still fail to meet practical application requirements [27].

The core crux of this chasm lies in the extreme complexity of solid–solid interfaces [28]. Unlike in liquid batteries, where electrodes and electrolytes can form intimate contact through wetting, all components in solid‐state systems are rigid solids. Atomic‐scale contact defects are sufficient to block ion transport pathways. More critically, aluminum ions (Al^3+^) possess high charge density, large ionic radius (∼ 54 pm), and strong polarizing ability, making their solid‐state transport intrinsically difficult [29, 30]. Any imperfection at the interface amplifies this difficulty by several orders of magnitude. Therefore, the development of SSABs is essentially a protracted war against interfaces, only when we understand and control these interfaces at the atomic scale can we transform SSABs from miracles in papers into practical devices.

To structure this complex problem, this review explicitly identifies the three core interfacial challenges facing SSABs, which constitute the fortresses that must be overcome to bridge the gap from materials to devices: (i) The triple predicament of contact, dendrites, and passivation for Al anode/solid electrolyte interphase (SEI) [31]. This interface is the most vulnerable link in SSABs. (ii) Multidimensional coupling from charge transport to structural stability for cathode/SEI [32]. The cathode‐side interface is equally fraught with peril. Most high‐performance cathode materials (e.g., transition metal oxides, sulfides, Prussian blue analogues) also suffer from poor physical contact with the solid‐state electrolytes [33, 34, 35], leading to low utilization of active materials. More importantly, the contact between two solid materials can form a space charge layer due to differences in chemical potential. (iii) The overlooked ion transport bottleneck for grain boundary issues within the solid‐state electrolyte [36]. Beyond the two electrode interfaces, the grain boundaries within the solid‐state electrolyte are equally crucial. In polycrystalline solid‐state electrolytes, grain boundaries are often slow channels for ion migration, with impedance potentially 2–3 orders of magnitude higher than within the grain interiors.

These three interfacial issues do not exist in isolation but form a complex system that is interconnected and dynamically evolving. For instance, degradation at the anode interface can exacerbate local current density inhomogeneity [37], promoting dendrite formation, and dendrite penetration may contaminate the cathode interface, and grain boundary defects within the electrolyte provide growth channels for dendrites. Therefore, a systemic mindset is essential for understanding and addressing these challenges. Faced with the aforementioned complex interfacial challenges [38], a vast body of research has proposed solutions from various angles. However, these works are often scattered across sub‐fields, lacking systematic integration and deep mechanistic analysis. Most reviews merely catalogue phenomena from the literature [39, 40], failing to reveal the physicochemical essence behind interfacial behavior and seldom addressing cross‐scale engineering practices from nanoscale design to macroscale device integration.

**FIGURE 1:**
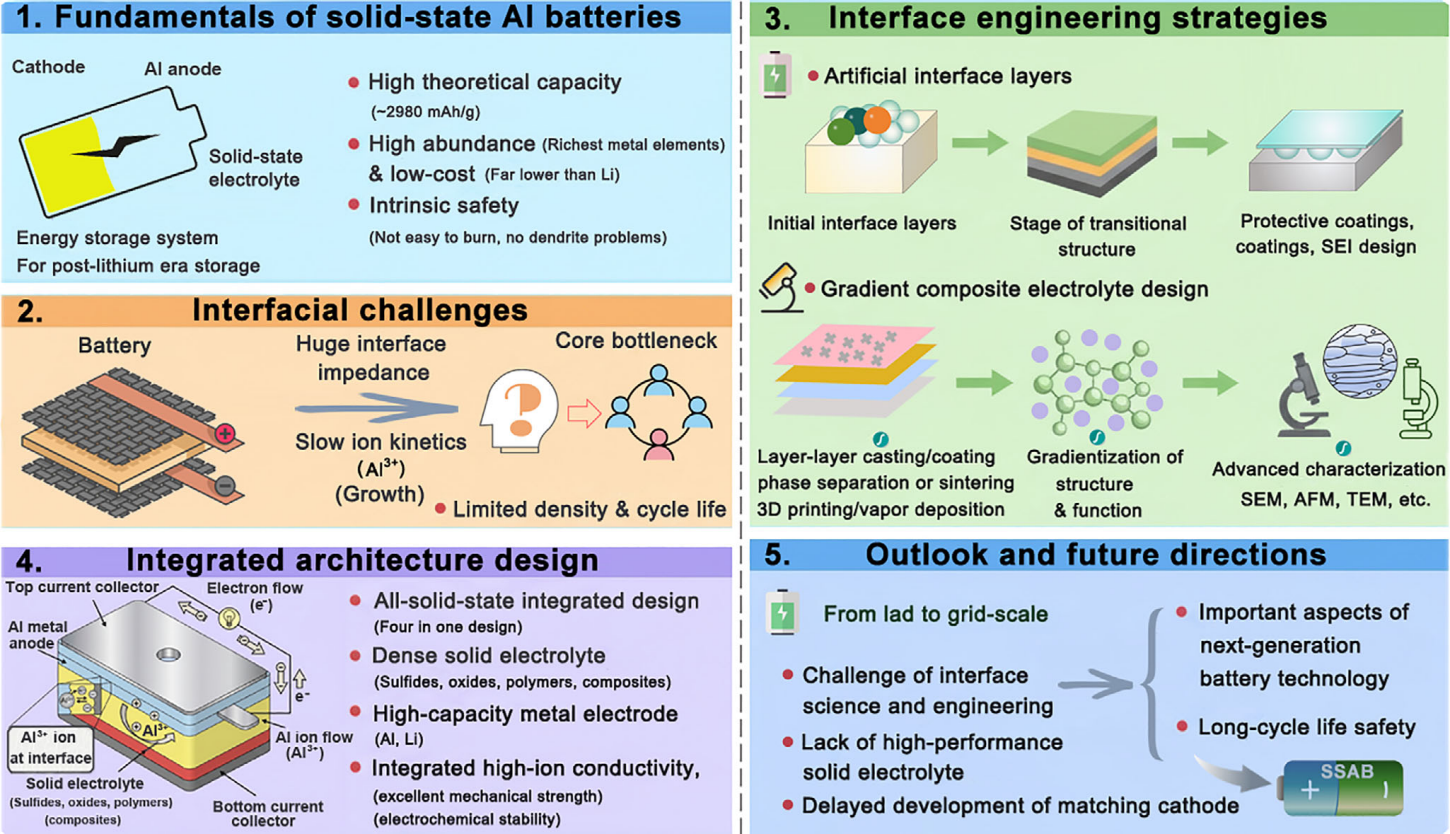
The framework of this study focuses on solid‐state aluminum batteries (SSABs).

This review aims to fill this gap, and the core question to be addressed is that in the SSAB system (Figure 1), how can cross‐scale interfacial engineering strategies be employed to synergistically resolve the solid–solid interface challenges at the anode/electrolyte, cathode/electrolyte, and within the electrolyte itself, ultimately realizing practical SSAB devices with high energy density, long cycle life, and intrinsic safety? Through a systematic response to this question, we hope to provide clear scientific guidance and an engineering blueprint for the rapid development of this emerging field, accelerate the transition of SSABs from laboratory concepts to industrial applications, and contribute key solutions to the energy storage revolution in the post‐lithium battery era.

However, it is important to clarify the relationship between SSABs and the extensive body of research on Li‐based solid‐state batteries that is referenced throughout this review. SSABs are an emerging field in its early stages, with a relatively limited publication record compared to Li‐based systems. Consequently, many of the characterization techniques, interfacial engineering strategies, and even conceptual frameworks employed in SSAB research are directly inherited or adapted from Li‐based solid‐state battery literature. This cross‐system borrowing is not indicative of a thematic drift, but rather reflects the intellectual and technical lineage of the field. In this review, we deliberately include relevant Li‐based studies for three purposes: (i) to provide the methodological origins of advanced characterization tools and interface design strategies now applied to SSABs, (ii) to enable comparative analysis that highlights the unique transport and stability challenges of Al^3+^ vs. Li^+^, and (iii) to document cross‐system conceptual transfers that demonstrate the generalizability of interfacial engineering principles. We have endeavored to clearly distinguish between Al‐specific findings and Li‐based precedents throughout the text, with the former serving as the central focus and the latter providing essential context and reference.

## 2 Physical and Chemical Roots of Interface Challenges in SSABs

### 2.1 Thermodynamics and Interface Stability

For SSABs, analyzing the interfacial stability between aluminum (Al) and various solid‐state electrolytes from a thermodynamic perspective provides the theoretical foundation for understanding interface failure mechanisms and designing stable interfaces [41]. Fundamentally, whether interfacial side reactions occur and the nature of their products is determined by the change in Gibbs free energy (*ΔG*) before and after the reaction [42]. However, the core thermodynamic criterion for interface stability lies in the propensity of Al to reduce the solid‐state electrolyte. The central ions in typical solid‐state electrolytes (such as S, P, O, and Cl) are usually in their highest or relatively high oxidation states. Consequently, when in contact with metallic Al, a strong reducing agent Al tends to reduce the electrolyte. The magnitude of this thermodynamic driving force is directly proportional to the potential difference between Al and the decomposition products of the solid‐state electrolyte.

A critical insight from this thermodynamic perspective is that alloy anodes (e.g., Al–Si, Al–Zr) possess electrode potentials higher than that of pure Al [43], thereby significantly reducing the driving force for electrolyte reduction. This alloying strategy, therefore, represents an important pathway for enhancing interface stability, a theme that recurs throughout the literature. Additionally, enhanced ionic diffusivity via phase engineering, the most direct kinetic benefit of alloying lies in the formation of fast ion‐conducting phases. In Al‐In alloys, for instance, the In phase is preferentially lithiated during initial charging, forming a LiIn phase that exhibits exceptionally high Li^+^ diffusivity [29]. This LiIn phase remains lithiated during subsequent cycles and serves as a 3D percolation network distributed throughout the Al matrix, providing continuous high‐diffusivity pathways for rapid ion transport [15]. Similarly, in Li‐Al alloys, the β‐LiAl phase exhibits Li^+^ diffusivity that is orders of magnitude higher than the α‐phase, enabling fast ionic conduction through the bulk electrode. This phase‐engineering approach fundamentally transforms the electrode from a kinetically limited system into one with built‐in fast ion transport channels. Furthermore, alloying elements with lower nucleation overpotentials, such as In, Bi, and Sn are preferentially lithiated or alloyed, creating surface layers that serve as catalytic sites for subsequent electrochemical reactions. These surface modifications lower the activation energy for interfacial charge transfer, as evidenced by reduced polarization and improved rate capability in alloyed anodes compared to pure Al [43].

To conduct an in‐depth analysis of the chemical and electrochemical stability of Al with various classes of solid‐state electrolytes (oxides, sulfides, halides, and polymers), and to discuss the thermodynamic driving forces of interfacial side reactions (including passivation layer formation), we critically examine selected prior studies.

**FIGURE 2:**
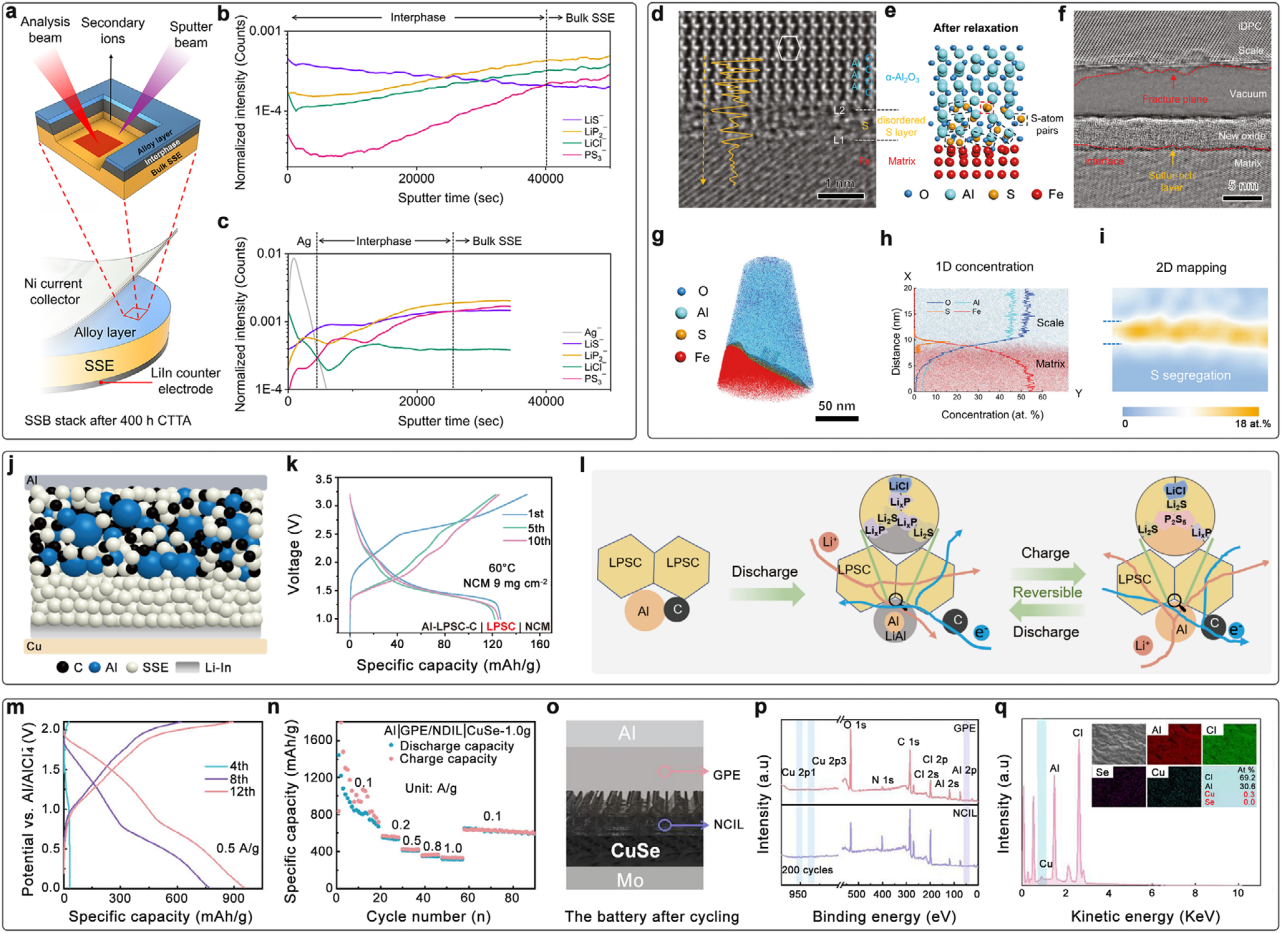
(a) ToF‐SIMS measurements were conducted on samples from SSB stacks subjected to 400 h of CTTA under 50 MPa stack pressure. (b, c) ToF‐SIMS depth profiles of the electrode/solid‐state electrolyte interface from SSB stacks with bare Ni and Ag‐coated Ni electrodes. Reproduced with permission [44]. Copyright 2025, American Chemical Society. (d) Filtered iDPC image and an intensity line profile taken across the scale‐matrix interface (orange line). (e) A simple oxide‐matrix interface, similar to (d), constructed by a DFT simulation. (f) iDPC imaging. (g) Atomic maps of all constituent elements from a 3D reconstruction. (h, i) 1D concentration and 2D mapping quantitative analysis of the oxide‐matrix interface. Reproduced with permission [45]. Copyright 2025, Springer Nature. (j) Schematic diagram of Li−In//Al half‐cells. (k) Representative charge−discharge curves of the Al‐LIC‐C//NCM622 full cell. (l) Schematic diagram of interfacial reaction in Al‐LPSC‐C|LPSC|Li−In half‐cells. Reproduced with permission [46]. Copyright 2025, American Chemical Society. (m) The GCD profiles of the Al|GPE/NCIL|CuSe battery at 0.5 A g^−1^. (n) The rate capability. (o) Schematic diagram of the Al|GPE/NCIL|CuSe battery after cycling. (p) The corresponding XPS spectra. (q) EDS spectra of GPE. Reproduced with permission [47]. Copyright 2025, Wiley‐VCH GmbH.

For instance, Jeong et al. [44] experimentally confirmed that Al alloy anodes can significantly suppress interfacial reactions with Li_6_PS_5_Cl. Notably, the driving force for the reaction is lower than that for lithium metal, and its growth mechanism is correlated with applied pressure (Figure 2a) and the mechanical properties of the constituent materials. By comparing the theoretical Gibbs free energy changes corresponding to the potential differences when aluminum (and its various alloy phases) [42] vs. lithium metal react with sulfide solid‐state electrolytes (e.g., Li_6_PS_5_Cl), the authors directly quantified the magnitude of the driving forces for interfacial reactions, including passivation layer formation (Figure 2b,c). While this study provides significant reference value, it is important to recognize that thermodynamics alone does not tell the full story.

Indeed, thermodynamic driving force is not the sole determining factor; kinetics and material microstructure are equally crucial. A compelling demonstration comes from Li et al. [45], who utilized atomic‐scale characterization techniques and density functional theory (DFT) calculations to systematically investigate the atomistic mechanism of oxide scale spalling in heat‐resistant alloys (Figure 2d–f). Based on these findings, they proposed an effective strategy to improve oxide scale adhesion through microalloying. Their research revealed that trace sulfur impurities segregate at the Al_2_O_3_/alloy interface and weaken interfacial bonding. Thermodynamic calculations were employed to analyze the driving forces for sulfur segregation and its inhibition by Zr microalloying (Figure 2g–i).

This study carries an important broader message: during high‐temperature oxidation, impurity segregation at interfaces can dramatically alter oxide film adhesion. By analogy, in electrochemical environments, applied stack pressure and the mechanical properties of electrode materials can significantly influence the rate and mode of side reactions by modifying the interfacial contact state [44]. It is noteworthy that this mechanism is not only applicable to the oxide scale/alloy interface but may also provide insights for addressing grain boundary brittleness caused by impurity segregation in other material systems, a point often overlooked in the battery literature.

Another representative study by Cui et al. [46] systematically investigated the electrochemical behavior of Al as an anode material in inorganic all‐solid‐state lithium batteries (Figure 2j). The core focus was the compatibility of the Al anode with two fundamentally different solid‐state electrolytes: a halide (Li_3_InCl_6_) and a sulfide (Li_5.4_PS_4.4_Cl_1.6_). Remarkably, these two systems exhibited entirely distinct interfacial side reaction mechanisms and product reversibility, a finding that underscores the electrolyte‐specific nature of interface design.

During electrochemical cycling (Figure 2k), Li_3_InCl_6_ continuously reduces to generate metallic indium, which accumulates on the surface and interface of the Al electrode, forming a thick In layer (∼ 30 µm). This layer impedes Li^+^ transport [4, 5], ultimately leading to battery failure. In stark contrast, in the sulfide electrolyte system, during the charging process, the reaction products reversibly transform into P_2_S_5_, forming a redox‐active interphase. This interphase undergoes reversible conversion (P_2_S_5_ ↔ Li_x_P + Li_2_S) in subsequent cycles, significantly enhancing the reversibility of the Li–Al alloying/dealloying process (Figure 2l). Moreover, the Al–LPSC–C electrode maintains structural stability during cycling, with no observable cracks or accumulated layers at the interface, achieving a Coulombic efficiency of up to ∼ 99%.

The mere existence of a thermodynamically predicted reaction does not necessarily lead to detrimental failure; if the reaction products form a reversible, ion‐conductive interphase, the interface can actually become beneficial for cycling. This distinction between irreversible degradation and reversible interphase formation is frequently blurred in simpler thermodynamic analyses.

Beyond SSABs, conventional aluminum batteries using chloroaluminate ionic liquid electrolytes suffer from well‐documented issues, including aluminum dendrite growth and the shuttle effect (dissolution of the CuSe cathode), which lead to rapid capacity decay and poor cycling stability. To address these persistent problems, Li et al. [47] proposed a strategy of introducing an ion/electron dual‐conductive interlayer between the gel polymer electrolyte and the CuSe cathode, which significantly enhanced the electrochemical performance of SSABs [19, 20].

Compared to the system without the N‐doped carbon interlayer (NCIL), the Al|GPE/NCIL|CuSe battery exhibited substantial improvements in rate capability (Figure 2m,n), cycling stability, and self‐discharge suppression. Importantly, the nitrogen‐doped carbon in the NCIL demonstrates a strong adsorption capacity for dissolved Cu and Se species, a finding verified by DFT calculations, where the N5 and N6 configurations show higher adsorption energy for AlCl_4_^−^ and Al_2_Cl_7_^−^ anions (Figure 2o–q). In summary, this multifunctional interlayer strategy simultaneously addresses the shuttle effect and dendrite growth while significantly enhancing capacity and cycle life. It provides a new approach and experimental foundation for the development of high‐safety, long‐life, high‐energy‐density solid‐state aluminum batteries.

In conclusion, the interfacial stability of Al anodes in solid‐state batteries is governed by a complex interplay of thermodynamics, kinetics, microstructure, and interfacial mechanics. While thermodynamic driving forces (ΔG) provide an essential first screening criterion, they are neither sufficient nor always predictive of actual cycling performance. As the contrasting cases of halide vs. sulfide electrolytes demonstrate, the reversibility and transport properties of reaction products often matter more than the mere presence or absence of a reaction. Furthermore, impurity segregation (as shown in oxide scale studies) and mechanical factors (stack pressure, material compliance) can substantially alter interface evolution factors that are frequently neglected in conventional electrochemical analyses. Moving forward, the design of stable Al|electrolyte interfaces will require integrated consideration of these multiple factors, moving beyond purely thermodynamic assessments toward a more holistic, multi‐physics framework.

### 2.2 Dynamics and Ion Transport Bottleneck

A central question in SSAB research concerns the chemical identity of the migrating aluminum species. The answer is electrolyte‐type‐dependent. In chloroaluminate‐based quasi‐solid electrolytes, charge carriers remain as AlCl_4_^−^/Al_2_Cl_7_^−^ complex anions, similar to liquid systems. In polymer electrolytes, Al^3+^ is partially dissociated and transported via coordination with polar groups (e.g., C═O, C─O) on polymer chains. In inorganic ceramics, Al^3+^ migrates as a genuinely free trivalent cation through interstitial sites or grain boundaries. This distinction has profound implications for interface engineering different electrolyte systems require fundamentally different optimization strategies.

Regarding the physicochemical origins of interfacial challenges in solid‐state aluminum batteries (SSABs) [19, 20], specifically the issues of kinetics and ion transport bottlenecks, three primary factors warrant consideration. The significant interfacial impedance arises from solid–solid point contacts [48], which create limited and discontinuous transport pathways. A high desolvation and migration energy barrier exists for Al^3+^ at the interface [49], stemming from the strong electrostatic interactions characteristic of trivalent ions. The inherently sluggish kinetics of multivalent ion transport [50] pose a fundamental limitation that distinguishes Al‐based systems from their Li counterparts. Elucidating the mechanisms underlying these aspects is therefore crucial for understanding and ultimately resolving the bottlenecks in kinetics and ion transport.

The sluggish kinetics in SSABs originate from the intrinsic properties of Al^3+^ itself [22]. Al^3+^ possesses an exceptionally high charge density and strong polarization ability due to its trivalent nature and small ionic radius (∼0.54 Å). These characteristics impose three fundamental constraints on solid‐state Al^3+^ transport: (i) high migration energy barrier in the lattice, requiring substantial structural rearrangements to break and reform multiple coordination bonds, (ii) high desolvation/decoupling barrier at interfaces, arising from strong coordination with electron‐donating species, and (iii) pronounced space charge layer effects, caused by strong electrostatic interactions with the local lattice [30]. These constraints are either absent or significantly milder in monovalent systems (Li^+^/Na^+^), underscoring why Li‐based strategies cannot be simply transplanted to SSABs.

From an engineering perspective, constructing an interlayer with both ionic and electronic conductivity between the electrolyte and electrode represents the most direct approach to address solid–solid point contact impedance. Furthermore, precisely modulating the van der Waals forces within the cathode material's interlayer and the interfacial charge state can effectively lower the solid‐state diffusion barrier for Al^3+^ at the interface [49]. These interfacial engineering strategies are key to improving overall kinetics.

However, it must be critically noted that traditional chloroaluminate ionic liquid electrolytes suffer from a triad of interrelated issues: aluminum dendrite growth, interface degradation, and high corrosiveness, all of which severely limit practical application. Moreover, in SSABs, the kinetic and ion transport bottlenecks are particularly acute: high solid–solid point contact resistance, high Al^3+^ migration energy barriers [29, 49], and slow multivalent ion transport kinetics collectively degrade performance.

**FIGURE 3:**
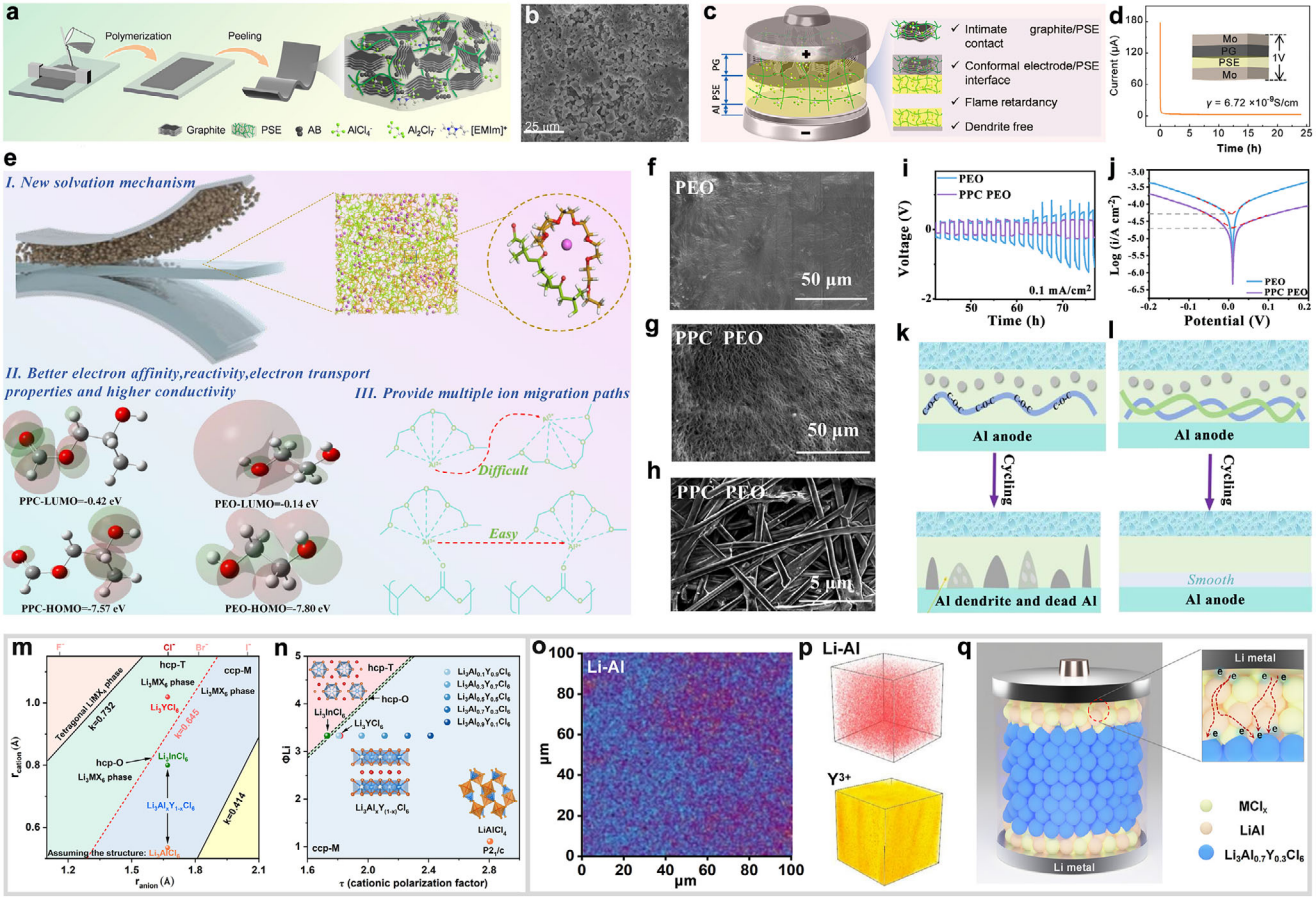
(a) Schematic illustration of the preparation process of the PG cathode. (b) SEM of the PG cathode. (c) Schematic diagram illustrating the structure for electrochemical performance and safety improvements of in situ polymerization on SABs. (d) i‐t response of the Mo/PG/PSE/Mo cell at an applied bias of 1.0 V. Reproduced with permission [51]. Copyright 2025, Wiley‐VCH GmbH. (e) Schematic of PPC‐PEO‐based solid electrolytes. (f–h) SEM of PEO SPE and PPC‐PEO BPE. (i) Local amplification plot of the cycling performance. (j) Tafel diagrams of PEO SPE and PPC‐PEO BPE. (k, l) Schematic of Al^3+^ deposition mechanism of PEO SPE and PPC‐PEO BPE. Reproduced with permission [52]. Copyright 2025, Wiley‐VCH GmbH. (m) Ionic radius rule of representative hcp‐T and ccp‐M type Li_a_MX_b_ halides. (n) Cationic polarization factor of LAYC electrolytes. (o, p) 2D diagram and 3D distribution of Li–Al alloy in the interfacial reaction layer obtained by TOF‐SIMS. (q) Scheme of the interfacial reaction mechanism of the Li|Li_3_Al_0.7_Y_0.3_Cl_6_|Li symmetric battery. Reproduced with permission [53]. Copyright 2025, Royal Society of Chemistry.

In response to these challenges, Li et al. [51] proposed a novel method for constructing SSABs through an in situ polymerization strategy (Figure 3a,b), which significantly enhances areal capacity, cycle life, and safety. A critical advantage of this approach is that in situ polymerization constructs a tightly contacted solid–solid interface between the polymer solid electrolyte (PSE) and the positive electrode, thereby avoiding the interfacial gaps commonly observed in conventional coating methods (Figure 3c). Furthermore, the PSE exhibits low activation energy (0.068 eV) and high ionic conductivity (Figure 3d), ensuring rapid migration of Al_x_Cl_y_^−^ anions. The anion transference number is 0.157, indicating cation‐dominated conduction, a desirable feature that facilitates the transport of Al^3+^‐related species.

In a complementary approach addressing electrolyte design, Pan et al. [52] proposed a novel polymer electrolyte system based on a blend of polypropylene carbonate (PPC) and polyethylene oxide (PEO) (Figure 3e), aimed at resolving core bottlenecks in aluminum‐ion batteries (AIBs), including difficult Al^3+^ migration [49], severe interfacial corrosion, and low room‐temperature ionic conductivity. By blending PPC with PEO and using a cellulose membrane as a support (Figure 3f,g), a blended polymer electrolyte was prepared.

The introduction of PPC brings multiple synergistic effects that warrant detailed examination. The carbonyl groups (C═O) of PPC form strong adsorption with Al^3+^ (−1.49 eV), partially replacing the ether oxygen coordination of PEO. This substitution decouples Al^3+^ anion pairs [50], increasing the concentration of free Al^3+^. The results are quantitatively impressive: the Al^3+^ transference number increased from 0.348 (for pure PEO) to 0.597, and the ionic conductivity near room temperature (∼ 35°C) reached 1.53 × 10^−4^ S/cm. Cyclic voltammetry (CV) curves further show that the redox peak potential difference decreased by 12.7% (from 0.714 to 0.623 V) (Figure 3i,j), indicating improved reversibility of the electrode reaction.

A critical mechanistic insight for PPC optimizes the Al^3+^ coordination structure, lowers the deposition energy barrier, and accelerates interfacial charge transfer. Through this PPC‐PEO blend design, the study achieves, for the first time, a triple synergistic enhancement of Al^3+^ transference number, ionic conductivity, and mechanical strength. Moreover, it provides a new theoretical framework, the strong adsorption fast dissociation mechanism, for understanding the transport mechanism of trivalent ions in polymer electrolytes (Figure 3k,l). This offers a viable electrolyte design strategy and experimental foundation for developing high‐safety, high‐energy‐density SSABs [19, 20].

Another representative study by Li et al. [53] proposed a halide solid‐state electrolyte, Li_3_Al_x_Y_1‐x_Cl_6_, with a high aluminum doping ratio. By modulating the Al^3+^/Y^3+^ ratio, the authors successfully constructed a three‐dimensional (3D) lithium‐ion transport network, significantly enhancing ionic conductivity and interfacial stability against metallic lithium. When Al^3+^/Y^3+^ = 0.7 (Figure 3m,n), Li_3_Al_0.7_Y_0.3_Cl_6_ exhibited an ionic conductivity of 1.05 × 10^−4^ S/cm at room temperature and an activation energy of 0.303 eV.

Time‐of‐flight secondary ion mass spectrometry (TOF‐SIMS) analysis (Figure 3o–q) revealed a particularly important finding: the formation of a Li–Al alloy (55.4, 72.8 eV) at the interface after cycling [54, 55], while Y^3+^ was not reduced. This indicates that the introduction of Al fundamentally alters the interfacial reaction pathway, forming a stable SEI layer [31, 32] rather than a deleterious degradation product.

Although this study focuses on Li‐ion conduction, its implications for Al‐based systems are profound. The primary solution strategy, constructing a 3D ion transport network through Al doping to lower the migration energy barrier, can be directly translated to the design of analogous 3D migration channels for Al^3+^, thereby overcoming the strong coordination and slow diffusion resulting from its high charge density. More broadly, this work provides significant insights for the electrolyte design of multivalent ion batteries (including aluminum batteries) [16], demonstrating that modulating crystal structure through heterovalent element doping, constructing multidimensional ion channels, and inducing the formation of stable interfacial phases are effective, transferable strategies to overcome kinetic and ion transport bottlenecks [29, 49].

In summary, the kinetic and ion transport bottlenecks in SSABs originate from three interrelated factors: solid–solid point contact impedance, high Al^3+^ migration energy barriers, and intrinsically sluggish multivalent ion transport kinetics. While these challenges are formidable, the studies reviewed above demonstrate that multiple orthogonal strategies can effectively address them. In situ polymerization [51] overcomes physical contact gaps by creating intimately bonded interfaces. Polymer blend engineering [52] tackles coordination chemistry by introducing strong‐adsorption functional groups that decouple ion pairs and lower migration barriers. Halide electrolyte design [53] offers a third pathway structural modulation through heterovalent doping to create 3D conduction networks and stable interfacial phases.

Regardless of the specific approach, successful mitigation of interfacial bottlenecks requires simultaneous consideration of physical contact, coordination chemistry, and crystal structure. Single‐factor optimizations are unlikely to succeed, instead, integrated, multi‐pronged strategies are necessary. Furthermore, the transferability of design principles between Li‐based and Al‐based systems, particularly the value of 3D ion transport networks and stable interfacial alloy formation, suggests a convergence of effective design strategies across multivalent battery chemistries.

### 2.3 Mechanical Stress and Morphology Evolution

In SSABs [47, 51], mechanical stress and morphological evolution are core issues affecting cycling stability and safety. Due to the significant volume changes accompanying the deposition and stripping processes of the aluminum anode, the interfacial contact state, stress distribution, and the nucleation and growth mechanisms of dendrites directly determine the battery's failure mode. It is well established that the molar volume of Al changes during alloying (e.g., formation of Li–Al alloy) or deposition processes. For instance, the volume expansion is approximately 96% when Al transforms into a LiAl alloy, while smaller than that of silicon (approximately 400%), it remains substantial and non‐negligible [54].

A critical distinction must be drawn between liquid and solid‐state systems. In solid‐state batteries, the rigid electrolyte cannot flow to accommodate volume changes as liquid electrolytes do. Consequently, fluctuations in interfacial contact pressure occur, often leading to the formation of interfacial voids or even complete loss of contact failure modes that are largely absent in liquid‐electrolyte systems.

The study by Li et al. [45] on oxide scale exfoliation provides valuable parallels. Interfacial sulfur segregation leading to a weakly bonded layer represents a classic example of mechanically induced interfacial failure. Similarly, in research by Cui et al. [46], interfacial side reactions of Al in the halide system Li_3_InCl_6_ generated an indium layer, causing deteriorated interfacial contact and hindered Li^+^ transport [53]. These examples underscore a general principle, the chemical reactions and mechanical degradation are deeply intertwined, and addressing one in isolation is unlikely to succeed.

In summary, stress regulation and interfacial engineering design are critical for overcoming performance bottlenecks in SSABs. Strategies such as constructing integrated interfaces through in situ polymerization [51], introducing rigid–flexible dual networks [56], and doping elements to modulate interfacial reactions can effectively mitigate failures caused by mechanical stress [57], thereby enhancing cycle life and safety.

Although high‐capacity alloy anodes (e.g., Si, Al, Sn) hold great potential [43], they suffer from severe electrochemical‐mechanical degradation under demanding conditions: high areal capacity (> 4 mAh cm^−2^) and low stack pressure (< 10 MPa). The main issues include large volume expansion, interfacial contact failure, localized stress concentration, and the risk of short circuits caused by creep of the anode material toward the cathode [3, 7]. A fundamental trade‐off thus exists: higher capacity inevitably brings greater mechanical instability, and no single material has yet resolved this dichotomy.

**FIGURE 4:**
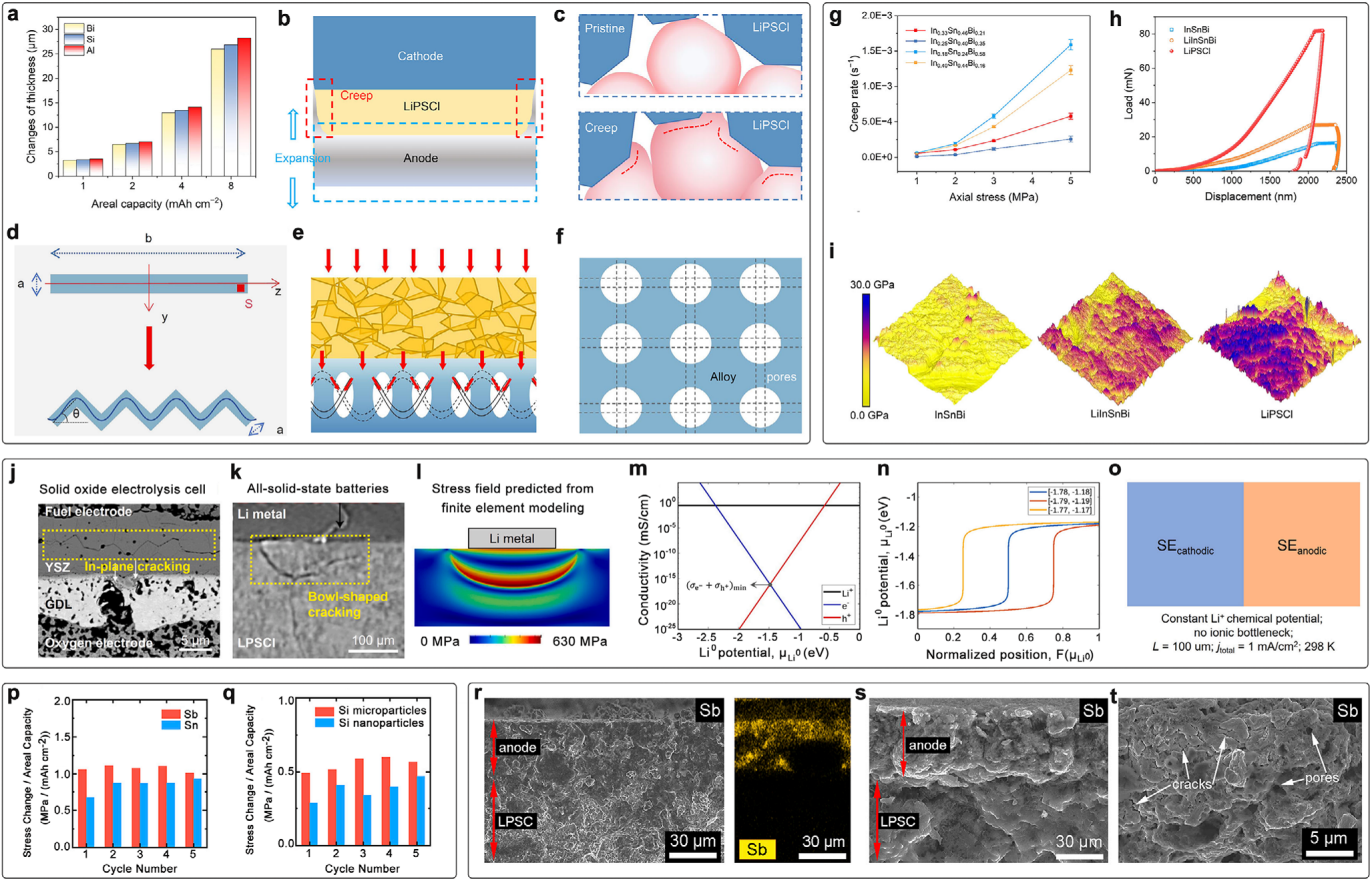
(a) Anode thickness variations across different areal capacities. (b) Schematic illustration of volume expansion mechanisms and resulting detrimental creep in high‐capacity alloy‐based anodes during cycling. (c) Time‐dependent evolution of interfacial creep behavior at the anode‐electrolyte interface. (d) Mechanical analysis comparing the moment of inertia between planar and curved titanium current collector architectures. (e) Stress distribution and pressure propagation pathways throughout the cell stack during cycling. (f) Longitudinal cross‐sectional view of the Ti‐InSnBi composite anode structure. (g) The relationship between the elemental ratio of the InSnBi alloy and its creep rate under the corresponding axial stress. (h, i) Load‐displacement curves and Young's modulus of pristine InSnBi, lithiated InSnBi, and LPSCl. Reproduced with permission [58]. Copyright 2025, Wiley‐VCH GmbH. (j) In‐plane cracking in yttrium‐stabilized zirconia (YSZ) in a solid oxide electrolysis cell. Reproduced with permission [59]. Copyright 2021, Elsevier. (k) Bowl‐shaped cracking in argyrodite (LPSCl) in an all‐solid‐state battery. Reproduced with permission [60]. Copyright 2021, Springer Nature. (l) von Mises stress field predicted by finite element modeling, showing a bowl‐shaped region with high stress reaching several hundred MPa. Reproduced with permission [61]. Copyright 2024, Elsevier. (m) Conductivities as a function of Li^0^ potential used for the calculations. (n) Li^0^ potential profile obtained through analytical calculations. (o) Schematic diagram of the model SE separator comprising two SEs. Reproduced with permission [62]. Copyright 2025, Elsevier. (p) Stress change normalized by areal capacity over five cycles for the Sb and Sn cells. (q) Stress change normalized by areal capacity over five cycles for the Si microparticle cell. (r–t) Cross‐sectional SEM images of Sb anode composites within cells after five cycles. Reproduced with permission [63]. Copyright 2021, Elsevier.

To effectively address these challenges, Sun et al. [58] proposed an innovative creep localization strategy (Figure 4a–f). By combining an InSnBi alloy (which exhibits high creep susceptibility) with a high‐moment‐of‐inertia titanium mesh, a composite anode structure was formed. Under low pressure, the alloy adaptively maintains good contact with the electrolyte through creep, while the titanium mesh with its rigid structure disperses localized stress (Figure 4g) and prevents excessive creep of the alloy toward the cathode (Figure 4h).

The results are noteworthy. This design enabled stable cycling under extreme conditions, the high areal capacity of 23.05 mAh cm^−2^, and low stack pressure of 3 MPa. The LiCoO_2_ || Ti–InSnBi full cell retained 81.6% of its capacity after 3000 cycles (2C) at 5.56 mAh cm^−2^. Ultimately, an energy density exceeding 350 Wh kg^−1^ was achieved, demonstrating excellent electrochemical‐mechanical stability (Figure 4i).

This creep localization strategy represents an elegant engineering compromise rather than a fundamental solution to volume change. By accepting controlled creep in a compliant alloy but confining it within a rigid scaffold, the design decouples the competing requirements of interfacial contact (which demands compliance) and dimensional stability (which demands rigidity). This principle may prove transferable to other alloy anode systems.

Additionally, transverse degradation refers to the accumulation of damage perpendicular to the direction of ionic current (Figure 4j) [59]. In its early stages, this phenomenon was observed in solid oxide cells as in‐plane propagating electrolyte cracks and oxygen bubble evolution at transverse grain boundaries. Recent studies have found that similar bowl‐shaped cracking (in‐plane cracking) occurs near the lithium metal anode in solid‐state batteries [60]. Importantly, this transverse damage precedes the longitudinal cracking that propagates along the ionic current direction and leads to eventual cell failure (Figure 4k).

The microscopic mechanism involves a general electronic bottleneck causing potential jumps of up to several electron volts. These jumps have a dual effect, on one hand, they drive atomic rearrangement and generate significant mechanical stress (Figure 4l) [61]. On the other hand, they result in a non‐uniform distribution of interfacial Li^0^. It is important to emphasize a subtle but crucial distinction, Li^0^ is not metallic lithium exsolved from the electrolyte lattice (Figure 4m,n), but rather a composite particle where Li^0^ is bound to an excess electron [62]. This distinction has important implications for how we interpret and model interfacial degradation.

Building on this foundation, to study the bilayer solid‐state electrolyte separator, Figure 4o establishes a model with equal‐thickness SE_cathodic_ and SE_anodic_ layers. This model is instrumental in analyzing ionic bottlenecks within the separator and at its interfaces, offering key insights into the mechanisms of interfacial contact failure and stress accumulation [57].

Another important study by Han et al. [63] systematically investigated the stress evolution mechanisms in alloy anodes during cycling in all‐solid‐state batteries [41, 64] and discussed their electrochemical behavior, material structure, particle size, and other relevant factors. A key finding is that alloy anodes cause significant stack pressure changes (∼ 1–2 MPa) during lithiation and delithiation (Figure 4p,q), substantially higher than those observed in graphite or LTO anodes. Moreover, the pressure curve exhibits nonlinear variation, with the most rapid pressure increase occurring at the end of charge/discharge. This indicates that the electrode structure can buffer expansion in the initial stages but becomes progressively constrained in the later stages, a stiffening behavior that accelerates degradation.

Post‐cycling scanning electron microscopy (SEM) revealed micron‐sized cracks and pores in the antimony (Sb) electrode (Figure 4r–t). These morphological changes lead to interrupted lithium‐ion transport paths [4, 5], localized stress concentration, increased interfacial impedance, and capacity decay. It can be seen that controlling particle size and optimizing electrode structure are key to mitigating stress accumulation and improving cycling stability.

In summary, mechanical stress and morphological evolution are not peripheral concerns but central determinants of cycling stability and safety in SSABs. Three interconnected failure mechanisms have been identified: (i) volume‐change‐induced interfacial contact loss, (ii) transverse (in‐plane) degradation preceding longitudinal failure, and (iii) stress accumulation leading to particle cracking and pore formation.

The studies reviewed above point toward several effective mitigation strategies. The creep localization approach [58] offers a pragmatic engineering solution that decouples compliance from dimensional stability. Understanding transverse degradation mechanisms [59, 60, 61, 62] reveals that electronic bottlenecks and potential jumps can drive damage in directions not previously anticipated, suggesting that electronic conductivity management may be as important as mechanical design. Finally, the work by Han et al. [63] demonstrates that particle size control and electrode architecture optimization are essential levers for managing stress accumulation.

The field has made significant progress in identifying and characterizing mechanical failure modes in SSABs. However, predictive models that couple electrochemical, mechanical, and morphological evolution remain in their early stages. Future advances will likely come from multi‐physics simulations that can predict stress distributions and failure initiation points, combined with experimental validation using in situ mechanical and imaging techniques. Until such predictive capabilities mature, empirical optimization as exemplified by the studies above will remain the primary pathway to mechanically robust SSABs.

### 2.4 Challenges and Insights of Characterization Techniques

Currently, solid‐state batteries are widely regarded as the future of energy storage technology due to their dual advantages of high energy density and safety. However, their core challenge lies in the complex instability of the solid–solid interface [65], which continues to hinder development like an impenetrable black box. This challenge is particularly acute in SSABs, which remain at the research frontier with significant unanswered questions. Nevertheless, recent literature has begun to reveal a systematic framework to overcome this difficulty [61, 62, 63].

The SSAB field finds itself in a paradoxical position. On one hand, it must draw upon established advanced characterization technology systems developed for lithium‐based systems [66]. On the other hand, it is crucial to recognize the significant shortcomings of dedicated technologies specifically for the aluminum system, shortcomings that cannot be simply filled by importing Li‐based tools. Therefore, adopting a novel collaborative research paradigm is not merely optional but essential.

From the perspective of technology adaptation, techniques developed for all‐solid‐state lithium‐ion batteries, such as neutron imaging [67], synchrotron radiation x‐ray tomography [68], focused ion beam scanning electron microscopy (FIB‐SEM) [69], and cryo‐transmission electron microscopy (cryo‐TEM) [70], have formed a complete imaging chain spanning from macroscopic distribution to atomic structure. These techniques have successfully revealed the microscopic mechanisms of lithium transport, dendrite growth, and interface failure. Importantly, their underlying principles and methodological frameworks can be directly adapted and utilized for aluminum battery research. This represents a significant opportunity for accelerated progress, provided the community actively pursues such cross‐fertilization.

**FIGURE 5:**
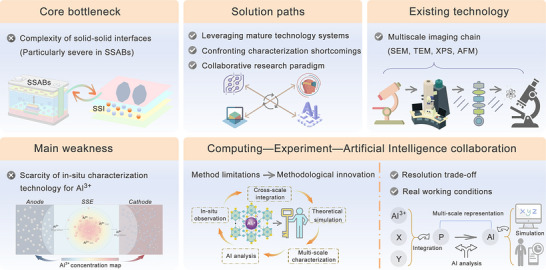
An overview of complexities surrounding the solid–solid interface in SSABs, shortcomings of existing characterization methods, and prospective strategies to address these challenges.

The complexity of solid–solid interface issues in SSABs (Figure 5) is multifaceted. The figure indicates that this complexity constitutes the core bottleneck of SSABs [31, 36, 65], primarily manifested in three interconnected domains: interfacial contact (physical intimacy and its evolution), stress evolution (mechanical deformation and accumulation), and side reactions (chemical and electrochemical degradation) between the negative electrode, solid electrolyte, and positive electrode. These three factors do not operate independently but rather interact synergistically, often in ways that are difficult to decouple experimentally.

Current characterization relies heavily on a multi‐scale imaging chain adapted from Li‐ion research. However, a critical shortcoming, indeed, a fundamental limitation of the current state of the art is the lack of in situ characterization techniques specifically designed for Al^3+^ [71]. This deficiency makes it exceedingly difficult to track Al^3+^ transport, interfacial reactions, and structural evolution in real time. Unlike Li^+^, which can be probed with relative ease using established methods, Al^3+^ presents unique challenges due to its higher charge density, slower kinetics, and the absence of suitable spectroscopic probes. This is not merely a technical inconvenience but a substantive barrier to mechanistic understanding.

To address this critical gap, a novel computation‐experiment‐artificial intelligence (AI) collaborative research paradigm is proposed. This approach emphasizes the deep integration of four pillars: theoretical simulations (providing mechanistic hypotheses and predictive frameworks), in situ observations (capturing real‐time dynamics), AI‐driven analysis (extracting patterns from complex, high‐dimensional data), and cross‐scale characterization (bridging atomic to macroscopic phenomena). The goal is to achieve multi‐scale, multi‐dimensional, and full‐operating‐condition understanding and control of interfacial processes [43, 44].

Traditional linear research workflows, where experiment follows simulation follows characterization are insufficient for the complexity of SSAB interfaces. Instead, an iterative, tightly coupled loop in which AI identifies patterns, simulations explain mechanisms, and experiments validate predictions offers a more promising path forward. This paradigm shift is not incremental but transformational.

Looking forward, future breakthroughs in in situ Al^3+^ characterization techniques stand out as the highest priority. Specifically, the development of operando spectroscopic methods (e.g., nuclear magnetic resonance, x‐ray absorption spectroscopy, or Raman scattering) with sufficient sensitivity and time resolution to track Al^3+^ coordination and transport at interfaces would fundamentally transform the field. Equally important is the construction of multi‐scale, multi‐modal collaborative research platforms that integrate complementary techniques. For example, combining synchrotron x‐ray tomography with electrochemical testing and AI‐based image analysis.

It is hoped that these advances will be key to achieving a deeper, predictive understanding of interfacial mechanisms and, consequently, the rational design of material systems for SSABs. Until such capabilities mature, the solid–solid interface will remain a partial black box. However, the collaborative paradigm outlined above offers the most viable pathway to illumination, one that leverages the strengths of computation, experiment, and AI in a synergistic, rather than sequential, manner.

## 3 Innovative Strategies for Interface Engineering

### 3.1 Construction and Protection of Al Anode/SSE Interface

In SSABs, the core challenge at the interface between the Al anode and the solid‐state electrolyte lies in a coupled chemo‐mechanical failure mechanism, a complex interplay of chemical degradation and mechanical instability that cannot be understood or addressed in isolation. This dual nature distinguishes SSABs from their lithium counterparts and demands integrated solutions.

The challenge manifests as pronounced interfacial chemical instability. The Al anode is prone to interfacial side reactions when in contact with solid electrolytes such as sulfides and halides. A critical comparison reveals starkly different behaviors depending on electrolyte chemistry. Research indicates that contact between Al and the Li_3_InCl_6_ halide electrolyte leads to continuous electrolyte decomposition [46], with metallic In accumulating at the interface. This In layer physically blocks Li^+^ transport channels [5, 72], causing rapid battery failure. In contrast, and this is a crucial distinction, sulfide electrolytes can form a redox‐active interphase with Al, which actually improves the reversibility of the alloying reaction rather than degrading it.

The chemical stability of the Al/electrolyte interface is not an intrinsic property of Al alone but rather a system‐level characteristic determined by the specific electrolyte chemistry. Some electrolytes are fundamentally incompatible with Al, while others can be engineered to form beneficial interphases. This electrolyte‐specific behavior underscores the importance of rational electrolyte selection and design.

The mechanical failure mechanism adds another layer of complexity. The Al anode undergoes significant volume expansion (∼ 96%) during the alloying and dealloying process [73], a magnitude that, while smaller than silicon, is still sufficient to cause serious mechanical degradation. This expansion leads to interfacial contact failure and stress concentration, particularly under constrained solid‐state conditions.

In situ atomic force microscopy (AFM) studies have revealed a more nuanced picture: Li–Al alloying is inherently non‐uniform, and the unalloyed Al phase exhibits sluggish kinetics [74]. This kinetic heterogeneity, combined with the high mechanical modulus of the LiAl phase, contributes directly to electrode cracking. The resulting failure mode can be termed kinetic‐mechanical coupled failure, where slow‐reacting regions become stress concentrators that initiate cracks, which in turn further impede reaction uniformity. This positive feedback loop significantly reduces the reversibility of the Al anode and represents a distinct failure mechanism not commonly observed in more homogeneous Li‐based systems.

Perhaps most fundamentally limiting, there is a critical lack of in situ characterization techniques specifically for Al^3+^ [71]. This is not merely a technical inconvenience but a substantive barrier to mechanistic understanding. Compared to the mature in situ characterization systems for lithium batteries, such as neutron imaging, synchrotron radiation x‐ray tomography, cryo‐electron microscopy, and operando x‐ray diffraction [67, 68, 69, 70], in situ tracking techniques for Al^3+^ remain severely underdeveloped. Consequently, it remains difficult to observe the transport, reaction, and structural evolution of Al^3+^ at the interface in real time. This blind spot hinders deep understanding of interfacial mechanisms and limits the field's ability to develop predictive models or rationally design improved interfaces.

Based on the above prominent issues, it is evident that incremental improvements are unlikely to suffice. Instead, a coordinated, multi‐pronged strategy is required. It is hoped that future efforts will focus on achieving synergistic regulation of the Al anode/solid‐state electrolyte interface from three interconnected dimensions:

1. Suppressing deleterious side reactions while promoting the formation of stable, ion‐conductive interphases. Strategies such as alloying regulation [75] and electrolyte design [76] are particularly promising.
2. Accommodating volume expansion and relieving stress concentration. Interlayer construction [77] and composite electrode architectures (e.g., the creep localization strategy discussed previously) can provide mechanical buffering.
3. Enabling real‐time management of interfacial evolution. This dimension remains largely unexplored for SSABs but could include adaptive stack pressure control or self‐regulating interlayer materials.

These three dimensions cannot be optimized independently. Rather, their interdependence demands an integrated approach. Combined with advanced characterization (particularly the development of in situ Al^3+^ probes) and theoretical simulations (multi‐physics models that couple chemical, mechanical, and transport phenomena), this integrated strategy will promote the practical application of SSABs. Future breakthroughs will likely come from tightly integrated efforts combining: (i) rational electrolyte and interlayer design to manage both chemistry and mechanics. (ii) Development of novel in situ characterization techniques specifically tailored to Al^3+^. And (iii) multi‐physics, multi‐scale modeling to predict and interpret interfacial behavior. Only through such a coordinated, cross‐disciplinary approach can the promise of SSABs be realized.

#### 3.1.1 Design of Artificial Interface Layers

In traditional electrolytes, Al^3+^ deposition is often hindered by competition from hydrogen evolution, leading to interfacial instability and the formation of byproducts such as Al(OH)_3_ [50, 78], which subsequently degrades battery lifespan. This issue is not unique to aluminum, similar parasitic reactions plague other multivalent and alkali metal systems. Therefore, solutions developed for one system often carry valuable lessons for others.

**FIGURE 6:**
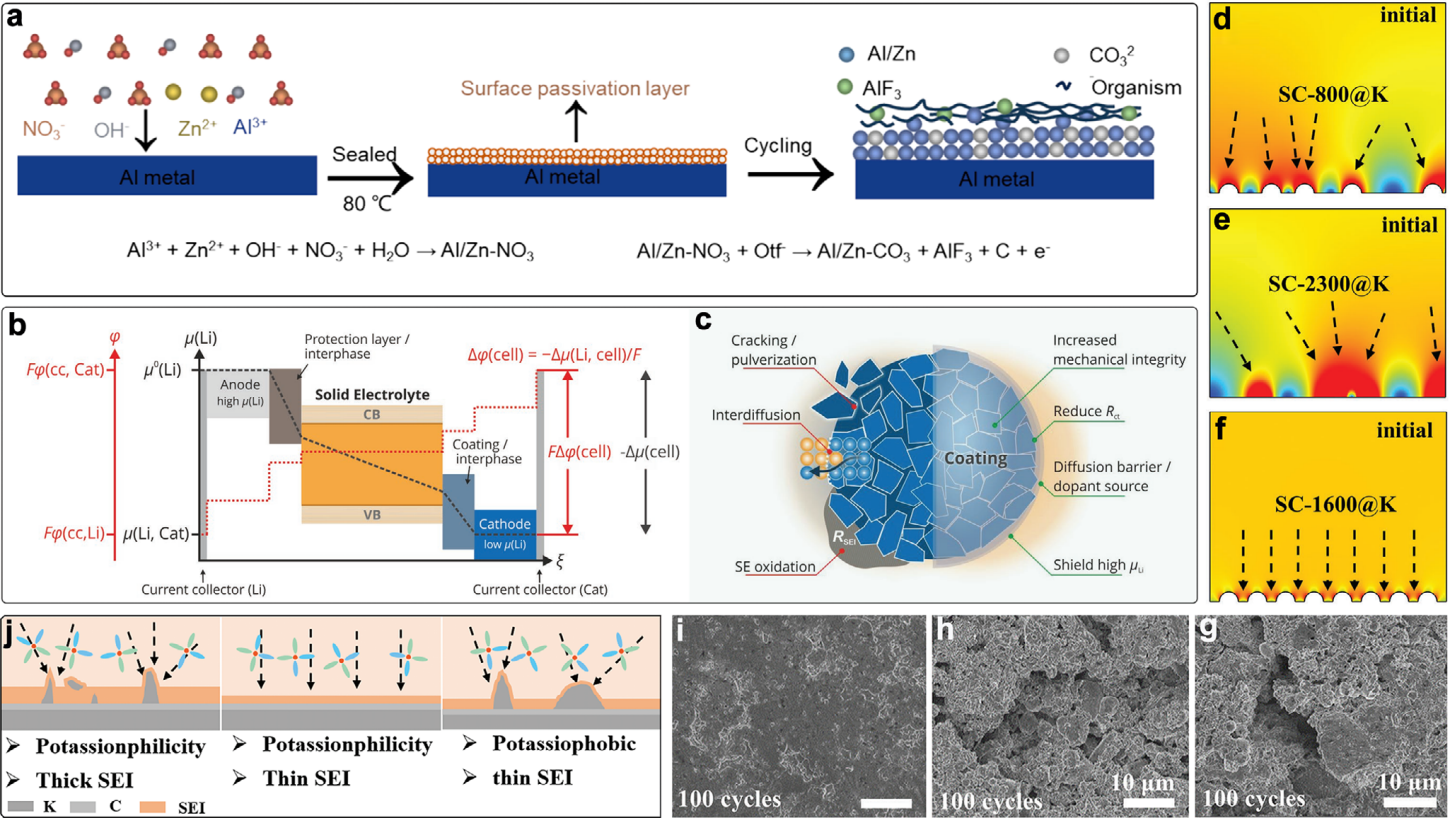
(a) Schematic diagram illustrating the synthesis of Al@SEI. Reproduced with permission [79]. Copyright 2024, Elsevier. (b) Potential profile in an SSB cell, highlighting the requirement for functional interphases at both the anode and the cathode. The variables *µ* and *ϕ* are the chemical potential of lithium (as a neutral component, in black) and the electric potential (in red), respectively. (c) Schematic illustrating the key issues arising at the interface between the cathode and the solid electrolyte during battery operation, as well as the benefits associated with the employment of a suitable cathode coating. Reproduced with permission [80]. Copyright 2019, Wiley‐VCH GmbH. (d) The 2D current density distribution simulated by COMSOL multiphysics of SC‐800@K. (e) SC‐2300@K. (f) SC‐1600@K. (g–i) The surface morphology of composite anodes after 100 cycles for SC‐800@K, SC‐2300@K, and SC‐1600@K. (j) The influence of adsorption energy and catalytic ability on K deposition and SEI formation. Reproduced with permission [82]. Copyright 2024, Wiley‐VCH GmbH.

To address this specific issue, Zhao et al. [79] constructed a bilayer SEI in situ on the aluminum electrode surface through hydrothermal and electrochemical activation methods (Figure 6a). This approach enhances interfacial chemical stability and suppresses side reactions such as hydrogen evolution and corrosion, thereby improving Coulombic efficiency and cycle life. Specifically, in a dilute Al(OTf)_3_–Zn(NO_3_)_2_ solution, a dense Al/Zn–NO_3_ passivation layer was formed on the Al surface via hydrothermal treatment, which was subsequently converted into a bilayer SEI through electrochemical activation.

The SEI constructed in this study is essentially an in situ‐formed artificial interfacial layer, and its design strategy aligns with common objectives shared across various artificial interfacial layer systems: guiding uniform metal deposition, suppressing side reactions, and enhancing interfacial stability. This design approach provides valuable insights for the future development of high‐performance metal batteries, such as Li and Zn, particularly in terms of structural design and functional integration of interfacial layers. The bilayer concept, where different layers serve distinct functions, represents a sophisticated design philosophy that transcends any single battery chemistry.

Recently, Culver et al. [80] systematically explored the functional design of surface coatings (i.e., artificial interfacial layers) on cathode active materials (CAM) in thiophosphate‐based all‐solid‐state batteries (Figure 6b,c). They emphasized the synergistic role of the coating in four key aspects: electronic insulation (to prevent parasitic reactions), ionic conduction (to enable charge transfer), mechanical compliance (to accommodate volume changes), and chemical stability (to withstand the operating environment).

Although their study focuses on the cathode, the design principles, material selection criteria, and performance evaluation methods for these interfacial layers are equally applicable to the design of artificial interfacial layers for metal anodes such as Al, Li, and Zn [81]. This is particularly relevant for suppressing side reactions, guiding uniform deposition, and enhancing cycling stability. The four‐function framework (electronic insulation, ionic conduction, mechanical compliance, chemical stability) provides a useful checklist for evaluating any artificial interfacial layer design, regardless of the target metal.

It is well known that potassium metal batteries (PMBs) offer advantages in high energy density and low cost, yet their practical application is hindered by issues such as uneven potassium deposition, dendrite growth, and instability of the SEI layer [31]. Conventional SEI layers are prone to cracking and reconstruction during cycling, leading to continuous electrolyte consumption, increased interfacial impedance, and shortened battery lifespan. The parallels to Al, Li, and Zn systems are unmistakable.

To address these challenges, Deng et al. [82] conducted a representative study in which they achieved dendrite‐free sodium/potassium metal anodes by regulating the balance between potassiophilicity and catalytic activity of an artificial interfacial layer. They designed a carbon layer with a locally ordered structure (SC‐1600) as the artificial interface layer (Figure 6d–f). The performance metrics are impressive: the SC‐1600@K symmetric cell demonstrated stable cycling for over 2000 h at 0.5 mA cm^−2^ and 0.5 mAh cm^−2^. Furthermore, the assembled PTCDA//SC‐1600@K full cell retained 78% of its capacity after 1500 cycles at 1 A g^−1^. Importantly, this strategy is also applicable to sodium metal batteries, significantly enhancing their cycling stability (Figure 6g–i).

By treating the precursor at different temperatures, the authors modulated the defect concentration within the carbon material, ultimately achieving uniform potassium deposition and the formation of a stable SEI (Figure 6j). This demonstrates that defect engineering tuning the density, and type of active sites is a powerful lever for controlling metal deposition behavior.

In summary, the design concept of artificial interfacial layers offers new insights for the development of metal anodes across multiple chemistries, including aluminum [51], lithium [55], and zinc [81].

More importantly, these insights are particularly valuable for guiding the suppression of dendrite growth, regulating SEI composition, and enhancing interfacial stability across diverse metal battery systems. The cross‐fertilization of design concepts between Al, Li, Na, K, and Zn systems still relatively rare in the literature, represents a significant opportunity for accelerating progress across the entire field of metal anode batteries.

#### 3.1.2 Alloying and Surface Modification

In SSABs, alloying and surface modification are two important interface engineering strategies aimed at reducing the nucleation overpotential of the aluminum anode and improving its wettability with the solid‐state electrolyte [15, 21], thereby enhancing the cycling stability and Coulombic efficiency of the battery. Alloying involves forming alloys of aluminum with other metals such as Zn, Sn, and Bi to regulate the electrochemical behavior of aluminum [83, 84, 85, 86]. Common examples of alloying include Al‐Zn alloys [87] and Al‐Sn alloys [88]. Surface modification, on the other hand, involves constructing an artificial interfacial layer on the aluminum surface through chemical or physical methods to improve its interfacial compatibility with the solid‐state electrolyte. Typical examples of surface modification include fluoride layers such as AlF_3_ [89], nitride layers such as AlN [90], carbon‐based coatings [91], organic/inorganic composite layers, and chemically treated layers.

More importantly, in SSABs, alloying and surface modification are two complementary interface optimization strategies. Alloying starts from the bulk material, regulating the deposition behavior and mechanical properties of aluminum. Surface modification optimizes the interfacial compatibility between aluminum and the solid‐state electrolyte by constructing an artificial interfacial layer. A synergistic design combining both strategies holds the potential to realize an aluminum anode system with low nucleation overpotential, high wettability, and long cycle life, laying the foundation for the practical application of SSABs [47, 51].

**FIGURE 7:**
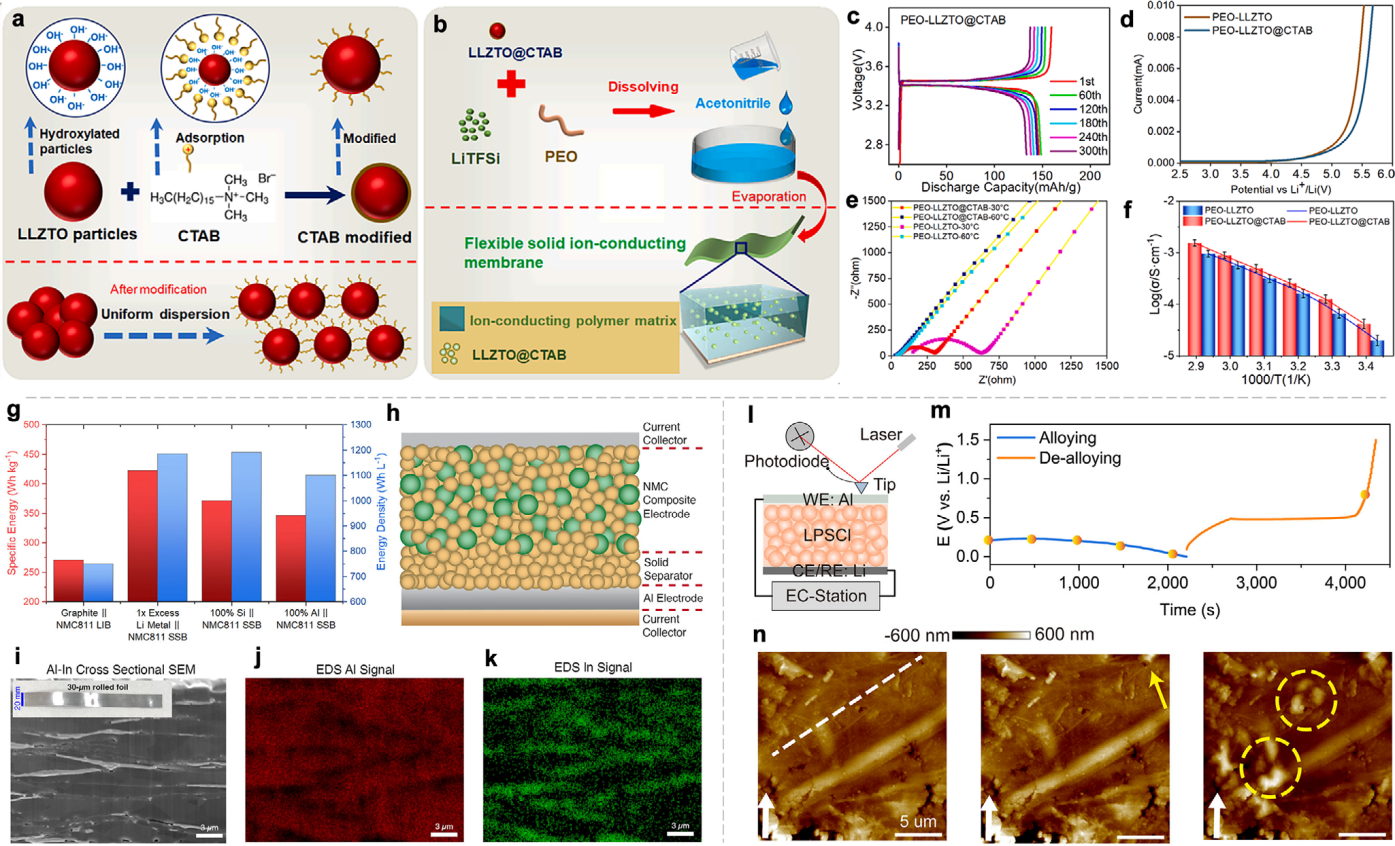
(a) Schematic illustration of the cationic surface‐modification mechanism. (b) PEO‐LLZTO composite solid‐state electrolyte (CSSE) membrane. (c) Charge/discharge curves. (d) LSV curves. (e) EIS curves. (f) Temperature dependence of the Li+ ion conductivity. Reproduced with permission [92]. Copyright 2023, Elsevier. (g) Theoretical stack‐level specific energy (Wh kg^−1^) and energy density (Wh L^−1^) comparison of a Li‐ion battery (LIB). (h) Schematic of a SSB with an aluminum‐based negative electrode. (i) Cryo‐FIB‐SEM image of a pristine Al–In alloy foil. (j) EDS map of the aluminum signal from a different SEM cross‐section. (k) EDS map of indium signal. Reproduced with permission [93]. Copyright 2023, Springer Nature. (l) Schematic in situ AFM cell diagram. (m) Charge–discharge curve of Al|LPSCl|Li using the in situ AFM cell at 25 µA cm^−2^. (n) In situ AFM experimental images collected at the orange dot in (m). Reproduced with permission [95]. Copyright 2025, Wiley‐VCH GmbH.

It is worth noting that although all‐solid‐state batteries have garnered significant attention due to their high energy density and enhanced safety, composite solid‐state electrolytes (CSSEs), which combine the flexibility of polymers (e.g., PEO) with the high ionic conductivity of inorganic fillers (e.g., LLZTO), face challenges. Specifically, LLZTO particles tend to agglomerate within the PEO matrix, leading to poor interfacial contact, reduced ionic conductivity, and issues such as lithium dendrite growth, which ultimately limit battery performance. To address this, Dong et al. [92] modified the surface of LLZTO inorganic particles using a cationic surfactant (CTAB) to improve their dispersion within the PEO‐based composite solid‐state electrolyte (Figure 7a,b), thereby enhancing the rate capability and cycling stability of all‐solid‐state batteries. In this study, CTAB adsorbs onto the negatively charged LLZTO surface through electrostatic interaction, forming a thin coating layer of approximately 5 nm. Following CTAB modification, the surface energy of the LLZTO particles is reduced, and their lipophilicity is enhanced, resulting in more uniform dispersion within the PEO matrix and improved interfacial contact. Full cell performance demonstrated that the LFP|PEO‐LLZTO@CTAB|Li cell retained 91.0% of its capacity after 300 cycles at 0.2C, with an initial discharge capacity of 146.6 mAh·g^−1^ and excellent rate capability (Figure 7c–f). Although this study focuses on modifying composite electrolytes for lithium metal batteries [21, 48], the surface modification strategy employed offers valuable insights for the application of aluminum anodes in solid‐state batteries. It holds particular promise for reducing nucleation overpotential, improving wettability, and suppressing dendrite growth.

Additionally, Liu et al. [93] investigated the use of aluminum foil anodes with a multiphase microstructure in all‐solid‐state LIBs. By introducing In through alloying, they enhanced the electrochemical performance of the aluminum anode, achieving high capacity, high rate capability, and long‐term cycling stability in all‐solid‐state batteries. In their study, the In phase preferentially lithiates during the initial charge, forming a LiIn phase. This LiIn phase remains lithiated during subsequent cycles, serving as a high lithium diffusion pathway that facilitates the reversible lithiation/delithiation of Al [94]. Under capacity‐limited testing conditions (2.1 mAh/cm^2^), the Al‐In battery cycled stably for 500 cycles (Figure 7g,h). Furthermore, Cryo‐FIB‐SEM analysis revealed that the Al‐In alloy maintained a dense structure after cycling, with the LiIn phase forming a 3D network distributed throughout the Al matrix (Figure 7i–k). This network provides rapid lithium diffusion pathways and suppresses lithium trapping. In contrast to liquid electrolyte batteries, the Al‐In anode in solid‐state batteries avoided pore formation and excessive SEI growth [31, 32]. In summary, this strategy provides a novel design concept for the application of aluminum anodes in all‐solid‐state batteries and offers valuable insights for the microstructural engineering of other alloy anodes such as Si and Sn.

In another important study, Liu et al. [95] investigated the lithiation/delithiation kinetics and mechanical failure mechanisms of aluminum anodes in all‐solid‐state batteries (Figure 7l). By introducing a Li_9_Al_4_ interfacial buffer layer through a co‐sintering surface modification strategy, they significantly enhanced the interfacial stability, reaction reversibility, and cycle life of the Al anode. The Li_9_Al_4_, characterized by its high lithium diffusivity and low modulus, serves as an interfacial buffer layer distributed on the surface of the Al matrix, forming 3D pathways for Li^+^ transport (Figure 7m) [4, 5]. Furthermore, in situ EC‐AFM revealed that unmodified Al formed uneven, wrinkle‐like protrusions after alloying, which failed to recover upon delithiation, indicating severe lithium trapping (Figure 7n). This strategy concurrently improved interfacial charge transfer, suppressed LPSCI decomposition, and alleviated mechanical stress.

In summary, the interface engineering landscape for aluminum anodes in solid‐state batteries has matured from simple trial‐and‐error approaches to systematic, mechanism‐guided design. Alloying [93] modifies bulk properties and creates percolation networks for fast ion transport. Surface modification [95] tailors the outermost interface, improving chemical compatibility and mechanical buffering. Surfactant‐based dispersion strategies [92], while developed for composite electrolytes, offer transferable principles for controlling interfacial chemistry.

No single strategy is sufficient. The most promising pathway forward involves synergistic integration of alloying (to optimize bulk transport and mechanical properties) with surface modification (to tailor interfacial chemistry and contact). Furthermore, the growing understanding that failure modes differ between liquid and solid electrolytes as evidenced by the absence of pore formation in solid‐state Al–In anodes, suggests that alloying strategies must be re‐evaluated and redesigned specifically for solid‐state systems, not simply imported from liquid‐electrolyte literature. This represents both a challenge and an opportunity for the SSAB community.

### 3.2 Optimization of Cathode/SE Interface

#### 3.2.1 Interface Coating and Buffer Layer

**FIGURE 8:**
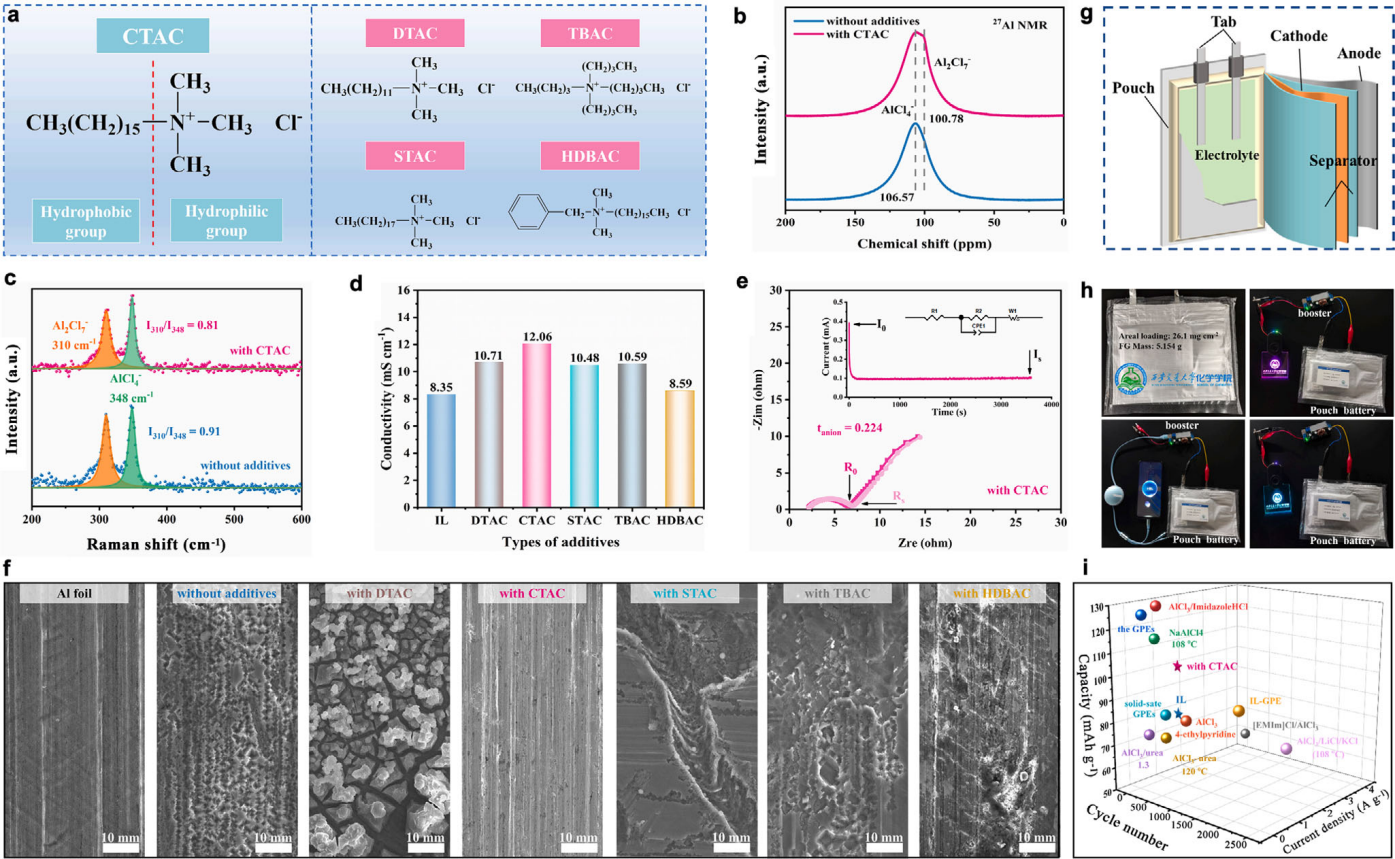
(a) Chemical formulas of DTAC, CTAC, STAC, TBAC, and HDBAC additives. (b) The 27Al NMR spectrum of the electrolyte without/with CTAC additives. (c) The Raman spectrum of the electrolyte without/with CTAC additives. (d) Conductivity of different electrolytes. (e) The EIS plot of the Al//Al symmetric battery using electrolyte with CTAC additives before and after polarization. The variation of current along with time during polarization was recorded at an applied voltage of 10 mV at room temperature. (f) SEM map of optimization of the electrode/electrolyte interface. (g) Diagram of the FG//Al pouch battery. (h) Photo of the pouch battery that power phones and light tags. (i) This work compares with the previously reported performance of RABs involving different electrolytes. Detailed references corresponding to this number are summarized in the original article. Reproduced with permission [96]. Copyright 2024, Elsevier.

Regarding the optimization of the cathode/solid electrolyte (cathode/SE) interface, Xie et al. [96] first proposed a dynamic molecular adsorption interface strategy. By introducing the cationic surfactant cetyltrimethylammonium chloride (CTAC) into an ionic liquid electrolyte (Figure 8a), they constructed a dynamic molecular adsorption interlayer to inhibit corrosion and dendrite growth on the aluminum anode, thereby enhancing the overall electrochemical performance of aluminum batteries. Unlike liquid‐phase interface regulation strategies that rely on surfactant adsorption via solvation diffusion, SSABs require solid‐state‐specific approaches that do not depend on mobile molecular species, a distinction that underpins the interface engineering strategies discussed.

Additionally, unlike static coatings or passivation layers that are permanently affixed to electrode surfaces, this dynamic adsorption layer forms and reforms in situ, adapting to changes in the electrochemical environment. This living interface represents a fundamentally different paradigm from conventional static protection strategies.

As the authors elucidate, the CTAC molecule featuring a hydrophobic long‐chain alkyl group and a hydrophilic quaternary ammonium group preferentially adsorbs onto the surfaces of both the Al anode and the FG (few‐layer graphene) cathode, forming a dynamic adsorption layer (Figure 8b,c). This bifunctional behavior is noteworthy, the same additive simultaneously protects both electrodes, albeit through distinct mechanisms.

For the Al anode, this adsorption layer inhibits corrosion and induces uniform Al deposition, addressing two of the most persistent challenges in aluminum batteries. For the FG cathode, it improves wettability and facilitates AlCl_4_^−^ ion transport [97]. Regarding the electrolyte, it regulates the AlCl_4_^−^/Al_2_Cl_7_^−^ concentration via Cl^−^, enhancing reaction kinetics. This triple‐functionality affecting anode, cathode, and bulk electrolyte simultaneously distinguishes dynamic adsorption strategies from single‐function interface modifications. The results are quantitatively impressive. The system demonstrated stable cycling for over 1200 h at 3 mA/cm^2^ and 1 mAh/cm^2^, with an average Coulombic efficiency of 99.99% (Figure 8d,e). Such high efficiency over extended cycling is rare for aluminum systems and approaches the standards of mature lithium‐ion technology.

Scanning electron microscopy (SEM) images showed that the CTAC adsorption layer leads to a more uniform distribution of current density and Al_2_Cl_7_^−^ (Figure 8f), suppressing the tip effect that typically drives dendrite nucleation and growth. Critically, the capacity increased by 10.5% (from 95 to 105 mAh/g) after 600 cycles, an unusual break‐in phenomenon where performance improves with cycling, likely due to gradual optimization of the dynamic equilibrium, whereas the pure ionic liquid battery began to decay after only 380 cycles (Figure 8i). High‐mass‐loading full cells (22.9 mg/cm^2^) and pouch cells also exhibited excellent rate capability and cycling stability (Figure 8g,h), demonstrating the scalability of this approach.

It is important to note that while this study primarily focuses on a dynamic adsorption layer constructed via electrolyte additives [98], its interface regulation strategy is highly consistent with the concept of constructing a mixed ion/electron conducting layer on the surface of cathode active particles. This conceptual alignment offers significant reference value, suggesting that dynamic adsorption interfaces may represent a generalizable approach rather than a system‐specific solution.

Therefore, this design strategy provides a novel approach for interface engineering in aluminum batteries and other metal batteries [99], holding particular importance for the development of multifunctional, dynamically adaptive interfacial buffer layers. The convergence of dynamic interface strategies [96], artificial interfacial (AI) layers [82, 89], and alloying approaches [93, 95] suggests that the future of interface engineering lies in integrated, multi‐functional, and adaptive systems rather than single‐purpose static coatings. For aluminum batteries in particular, dynamic molecular adsorption offers a promising pathway to overcome the persistent challenges of dendrite growth, interfacial corrosion, and poor cycling stability that have long hindered practical development.

It is worth emphasizing that while the dynamic molecular adsorption strategy demonstrated in [96] is based on an ionic liquid electrolyte system, its underlying interfacial regulation principles, particularly the formation of an adaptive molecular layer that homogenizes current distribution, suppresses tip effects, and enhances interfacial wettability, represent a generalizable paradigm rather than a system‐specific solution. Indeed, the authors explicitly note that this strategy can provide theoretical reference and practical guidance for interface modification and adjustment of other batteries electrolyte. In the context of SSABs, where similar challenges of non‐uniform ion flux, dendrite nucleation, and poor solid–solid contact persist, this dynamic interface concept offers valuable design inspiration. As will be discussed in subsequent sections, strategies such as surfactant‐modified ceramic fillers in composite electrolytes [92] and in situ polymerized integrated interfaces can be viewed as extensions of this adaptive interface philosophy to the solid‐state regime [51], demonstrating the cross‐system relevance of interfacial engineering principles.

#### 3.2.2 Integrated Cathode Electrode Electrolyte Structure

In SSABs, the integrated cathode‐electrolyte structure directly addresses the twin bottlenecks of high interfacial impedance and poor ion transport [100, 101]. Rather than simply pressing discrete layers together, an approach inevitably plagued by interfacial voids, this paradigm constructs a composite cathode with continuous ion conduction pathways. Two fundamentally distinct fabrication routes have emerged, the in situ polymerization [51, 102] and cold sintering [103]. The former generates a solid electrolyte directly within the cathode material via polymerization, achieving molecular‐scale interfacial contact. The latter applies pressure at moderate temperatures to densify and co‐sinter electrolyte and electrode components [62, 64], forming an intimately bonded, continuous transport network. While both reduce interfacial resistance and enhance cycling stability, their applicability depends critically on the material system, polymer‐based vs. ceramic, a distinction often glossed over in the literature.

**FIGURE 9:**
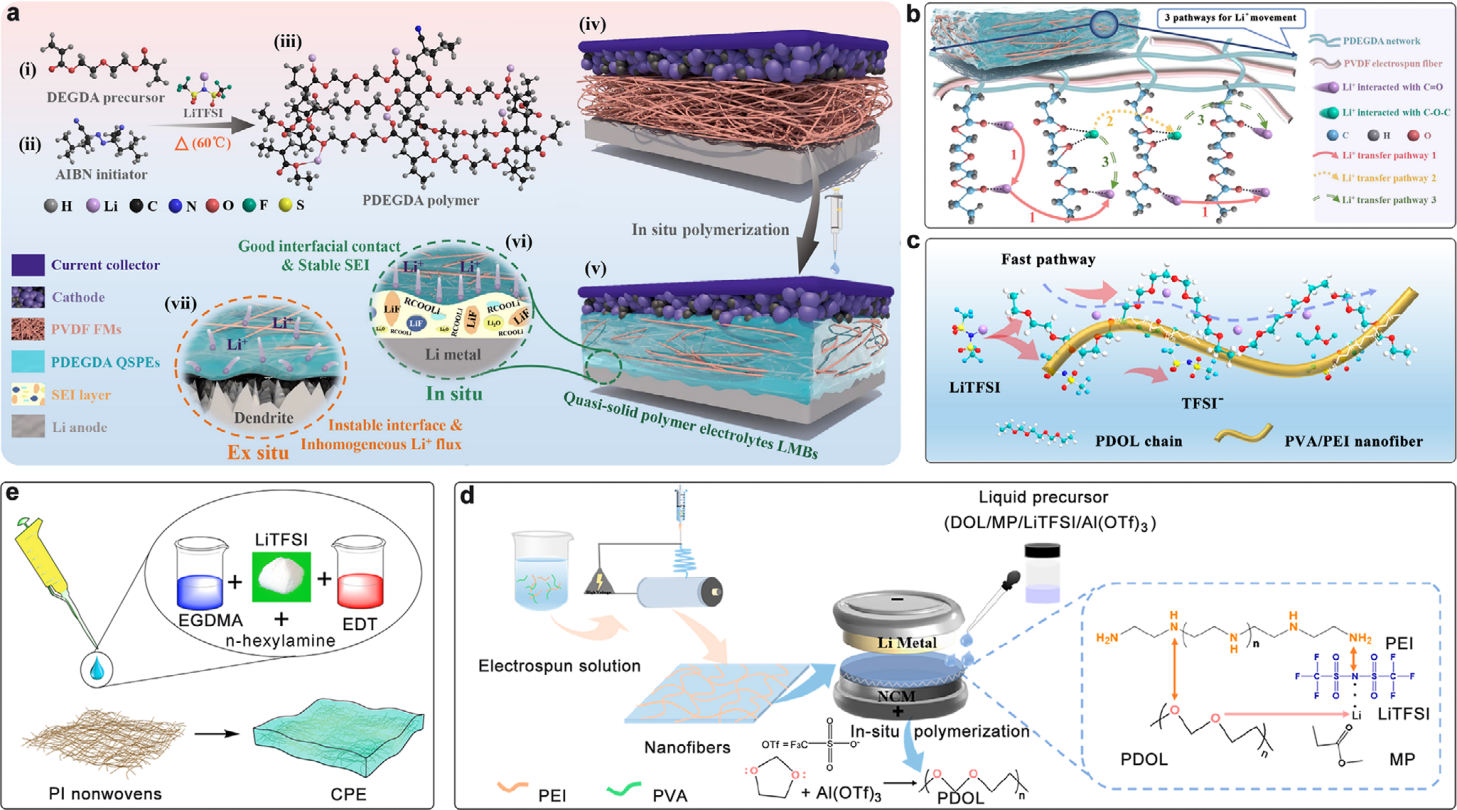
(a) Schematic illustration of the quasi‐solid‐state polymer LMBs prepared by in situ polymerization, which demonstrates the improvement of the interfacial contact by in situ preparation. (b) Schematic illustration of multiple transport pathways for Li^+^ in PDEGDA/PVDF FMs QSPEs. Reproduced with permission [104]. Copyright 2023, American Chemical Society. (c) Schematic illustration of the Li^+^ transfer pathway in QSE@PVA/PEI. (d) Preparation of QSE@PVA/PEI through in situ polymerization. Reproduced with permission [105]. Copyright 2023, American Chemical Society. (e) Preparation diagram of the P(EGDMA‐EDT)‐based CPE. Reproduced with permission [106]. Copyright 2021, American Chemical Society.

Recently, Lin et al. [104] employed in situ thermally induced polymerization to prepare a quasi‐solid polymer electrolyte (Figure 9a). The study addresses a well‐recognized dilemma, solid polymer electrolytes (SPEs) offer safety and processability, but suffer from low ionic conductivity and poor interfacial contact. The innovation lies in polymerizing diethylene glycol diacrylate (DEGDA) within a pre‐electrospun poly(vinylidene fluoride) (PVDF) framework. The resulting PDEGDA/PVDF composite achieves room‐temperature conductivity of 1.41 × 10^−4^ S/cm and a Li^+^ transference number of 0.454, attributed to C═O and C─O coordination with Li^+^ (Figure 9b). While these values represent incremental improvement over pristine polymer electrolytes, they remain substantially inferior to liquid or ceramic systems. The true contribution lies not in the absolute conductivity but in demonstrating that electrospun frameworks provide mechanical integrity without sacrificing ionic transport, a structural design principle transferable to SSABs.

In addition, Zhang et al. [105] developed a low‐temperature quasi‐solid electrolyte using in situ ring‐opening polymerization of 1,3‐dioxolane (DOL) within a PVA/PEI framework to form PDOL (Figure 9c). The resulting QSE@PVA/PEI electrolyte achieves a thickness of ∼ 21 µm and tensile strength of 12.1 MPa. A mechanistically interesting feature is the hydrogen bonding between PDOL and the framework (Figure 9d), which disrupts ordered PDOL chain packing and enhances segmental motion, thereby improving ionic conductivity at low temperatures. Therefore, this disordering by design strategy cleverly exploits hydrogen bonding for dual purposes (mechanical reinforcement and conductivity enhancement). However, the study's focus on low‐temperature performance, while valuable, raises an unanswered question that how does this same mechanism affect high‐temperature dimensional stability? Such trade‐offs are rarely discussed.

Similarly, Xu et al. [106] explored an all‐solid‐state polymer electrolyte based on thioether bonds, employing thiol‐Michael addition click reaction to polymerize ethylene glycol dimethacrylate (EGDMA) and 1,2‐ethanedithiol (EDT) within a polyimide (PI) nanofiber framework (Figure 9e). The resulting P(EGDMA‐EDT) electrolyte delivered 140.6 mAh/g at 0.05C with 96.2% capacity retention after 100 cycles at room temperature, and maintained stable cycling at 55°C. The pouch cell remained functional after disassembly, a pragmatic safety demonstration. The key mechanistic insight is the use of weak S–Li^+^ coordination to enhance ion transport, contrasting with the stronger O–Li^+^ coordination in conventional ether‐based electrolytes. However, while the weaker coordination lowers the hopping barrier, it may also reduce the total number of available coordination sites, creating a fundamental trade‐off between mobility and carrier concentration. This trade‐off is rarely addressed explicitly but is central to the rational design of polymer electrolytes for multivalent systems like SSABs.

Collectively, these three studies [104, 105, 106] demonstrate that in situ polymerization within reinforcing frameworks offers a versatile platform for integrated cathode‐electrolyte structures across diverse polymer chemistries thermally initiated free‐radical [104], ring‐opening [105], and click [106] polymerization. Each addresses a distinct operating regime: room‐temperature, low‐temperature, and high‐temperature performance, respectively. However, several critical observations warrant mention. All reported conductivities remain modest compared to ceramic or liquid electrolytes, highlighting that integration alone does not solve the intrinsic transport limitations of polymer systems. The mechanical properties of the composite electrolytes, crucial for accommodating volume changes, are often reported but rarely correlated with long‐term cycling stability. And most importantly for SSABs, all studies focus on Li^+^ transport; the extension to Al^3+^ with its higher charge density and slower kinetics is non‐trivial and cannot be assumed. Nevertheless, the integrated design principles (framework reinforcement, in situ formation, functional group engineering) provide valuable, transferable guidance for developing SSABs [18] where interfacial challenges are even more severe than in lithium systems.

### 3.3 Optimization and Interface Adaptation of Solid Electrolytes

#### 3.3.1 Composite Electrolyte Design

Notably, in the design of composite electrolytes for SSABs (and solid‐state metal batteries more broadly), the core purpose of constructing gradient or composite structures, such as polymer‐ceramic configurations [107], is to reconcile the inherent trade‐offs among ionic conductivity, mechanical strength, and interfacial contact. In these structures, a polymer serves as the continuous matrix, with inorganic ceramic fillers (e.g., LLZO, LATP particles or nanowires) dispersed within [108, 109, 110]. This design primarily leverages the flexibility of the polymer for processable film formation, while the dispersed ceramic phase enhances ionic conductivity and increases the mechanical modulus. In polymer‐in‐ceramic structures [107], although the rigid ceramic constitutes the main body, the surface in contact with the electrode is typically the polymer. The compliance and viscoelasticity of the polymer allow it to conform intimately to the electrode surface, filling its irregularities and thereby transforming solid–solid point contacts into area contacts, which significantly reduces interfacial impedance. Furthermore, ceramic electrolytes [111], especially oxides [112], often exhibit poor wettability toward lithium metal anodes and are prone to side reactions. Introducing or constructing a polymer layer on the ceramic surface physically isolates the ceramic from direct electrode contact, mitigating side reactions and stabilizing the interfacial chemical environment. Additionally, this approach is crucial for enhancing mechanical flexibility. For instance, ceramic‐polymer structures can overcome the inherent brittleness of ceramics [113]. It also enables the use of a ceramic framework to build long‐range, continuous expressways for fast ion transport, with the polymer filler acting as a bridge connecting the framework to the electrodes, forming a continuous 3D transport network that improves ion conduction pathways. In summary, constructing gradient structures, such as a ceramic‐rich side contacting the cathode and a polymer‐rich side contacting the lithium anode [107, 113], can further optimize interfacial compatibility, representing a key strategy for achieving high energy density and long cycle life in solid‐state batteries.

**FIGURE 10:**
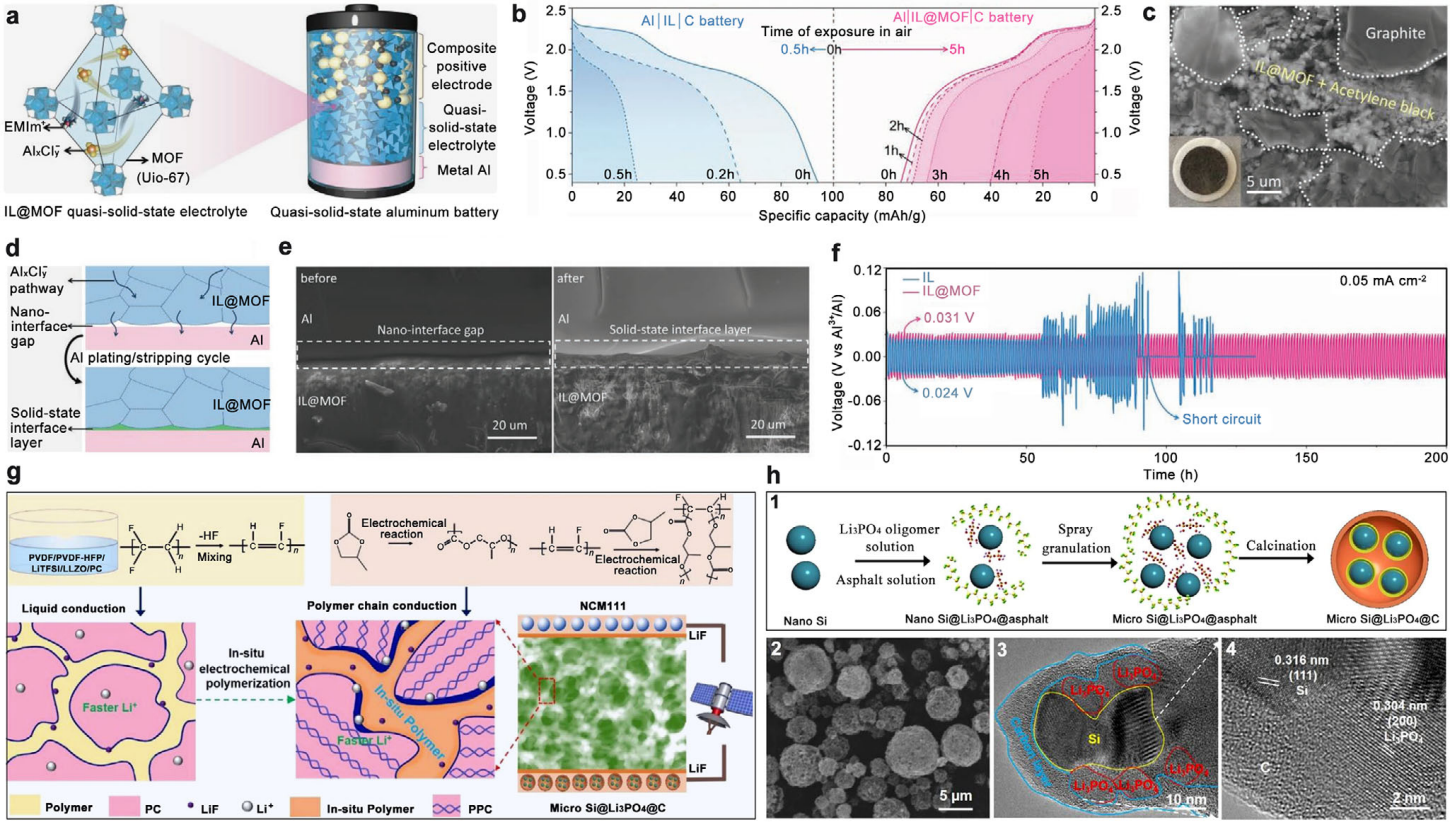
(a) Schematic illustration of the magnified internal structures in the IL@MOF electrolyte and the architecture of the quasi‐solid‐state aluminum battery. (b) The discharge curves of Al|IL|C and Al|IL@MOF|C batteries under the condition of exposure in air for different time scans. (c) SEM morphology of the composite positive electrode. (d) Schematic illustration of ion transport and IL@MOF electrolyte/Al metal interface changes before and after Al plating/stripping cycles. (e) SEM images of the IL@MOF electrolyte/Al metal interface before and after Al plating/stripping cycles. (f) Voltage profiles for the Al|IL|Al and Al|IL@MOF|Al cells at a current density of 0.05 mA cm^−2^. Reproduced with permission [114]. Copyright 2022, Wiley‐VCH GmbH. (g) Schematic diagram of SSBs. (h) Structure of Micro‐Si@Li3PO4@C and electrochemical properties in SSBs. Reproduced with permission [115]. Copyright 2021, Elsevier.

Recently, Huang et al. [114] investigated a quasi‐solid electrolyte based on a metal–organic framework (MOF) encapsulating ionic liquid (IL) to address issues in traditional liquid aluminum batteries, such as the high hygroscopicity, poor stability, and susceptibility to gas generation of ionic liquids. In this study, the IL ([EMIm]Cl: AlCl_3_ = 1:1.3) was encapsulated within a zirconium‐based MOF (Uio‐67) to form an IL@MOF composite quasi‐solid electrolyte (Figure 10a). The porous structure of the MOF (pore size 1.0–1.3 nm) accommodates IL ions (Al_x_Cl_y_^−^, EMIm^+^) while providing a physical barrier against moisture ingress. The electrolyte exhibits a room‐temperature ionic conductivity of 7.5 × 10^−4^ S cm^−1^, an activation energy of 165 meV, and an electrochemical stability window of 2.475 V (Figure 10b,c). The anionic transference number increased from 0.18 for the neat IL to 0.33, as the MOF pores restrict EMIm^+^ migration while facilitating Al_x_Cl_y_^−^ transport (Figure 10d). Furthermore, when paired with a graphite cathode, it delivered a discharge capacity of 75 mAh g^−1^ at a high areal loading of 9 mg cm^−2^. After 2000 cycles, the capacity remained at 62 mAh g^−1^ with a Coulombic efficiency > 98% (Figure 10f). The battery could still power an LED even after exposure to air for 2 h or under flame combustion conditions, demonstrating exceptionally high safety and stability. More importantly, within the composite cathode, IL@MOF particles were uniformly mixed with graphite and conductive carbon, forming a stable solid–solid interface between the electrolyte and the electrode (Figure 10e) [28]. This also created a 3D continuous ionic/electronic conductive network, significantly reducing interfacial impedance.

Additionally, Pan et al. [115] investigated a composite polymer electrolyte with high ionic conductivity and its application in matching with silicon anodes, aiming to promote the practical implementation of solid‐state batteries. This study constructed a 3D porous composite polymer electrolyte (3D‐PPLLP‐CPEs) (Figure 10g), composed of PVDF/PVDF‐HFP, LiTFSI, LLZO ceramic filler, and propylene carbonate (PC) plasticizer. The polymer and ceramic synergistically establish continuous ion conduction pathways.

A distinctive feature is that during the initial discharge process, PC underwent electrochemical in situ polymerization with PVDF/PVDF‐HFP (Figure 10h), while maintaining high ionic conductivity (3.3 × 10^−4^ S/cm at room temperature). Furthermore, the in situ polymerization eliminated free PC solvent, while the resulting PPC copolymer formed an intimate solid–solid interface with the electrode materials, significantly reducing interfacial impedance. However, this approach cleverly uses the plasticizer's initial presence to enhance conductivity during processing and formation, then eliminates it via polymerization to avoid the long‐term stability issues associated with residual solvent, a best of both worlds strategy.

In summary, this research [115] achieved a combination of high ionic conductivity, excellent interfacial contact, and good mechanical flexibility by constructing a 3D porous composite electrolyte based on PVDF/PVDF‐HFP + LLZO + PC. By utilizing in situ polymerization to eliminate free solvent and generate a LiF interfacial layer, it successfully matched with high‐capacity silicon anodes [116], providing new insights for the practical application of solid‐state batteries [68, 69, 76].

Several design principles emerge from these studies that are directly transferable to aluminum systems. Nanoconfinement (via MOFs or porous frameworks) can selectively modulate ion transport and protect against environmental degradation [114]. In situ polymerization within porous scaffolds eliminates free solvent while maintaining high conductivity and creating intimate interfaces [115]. Furthermore, gradient architectures enable spatial optimization of interfacial properties, acknowledging that a single composition rarely satisfies both cathode and anode requirements [107, 113]. However, the field must critically address whether the added complexity of such multi‐component, gradient structures is justified by performance gains, a question that becomes particularly acute for SSABs, where the baseline performance of simpler systems remains far from practical targets.

#### 3.3.2 Exploration of New Electrolytes

**FIGURE 11:**
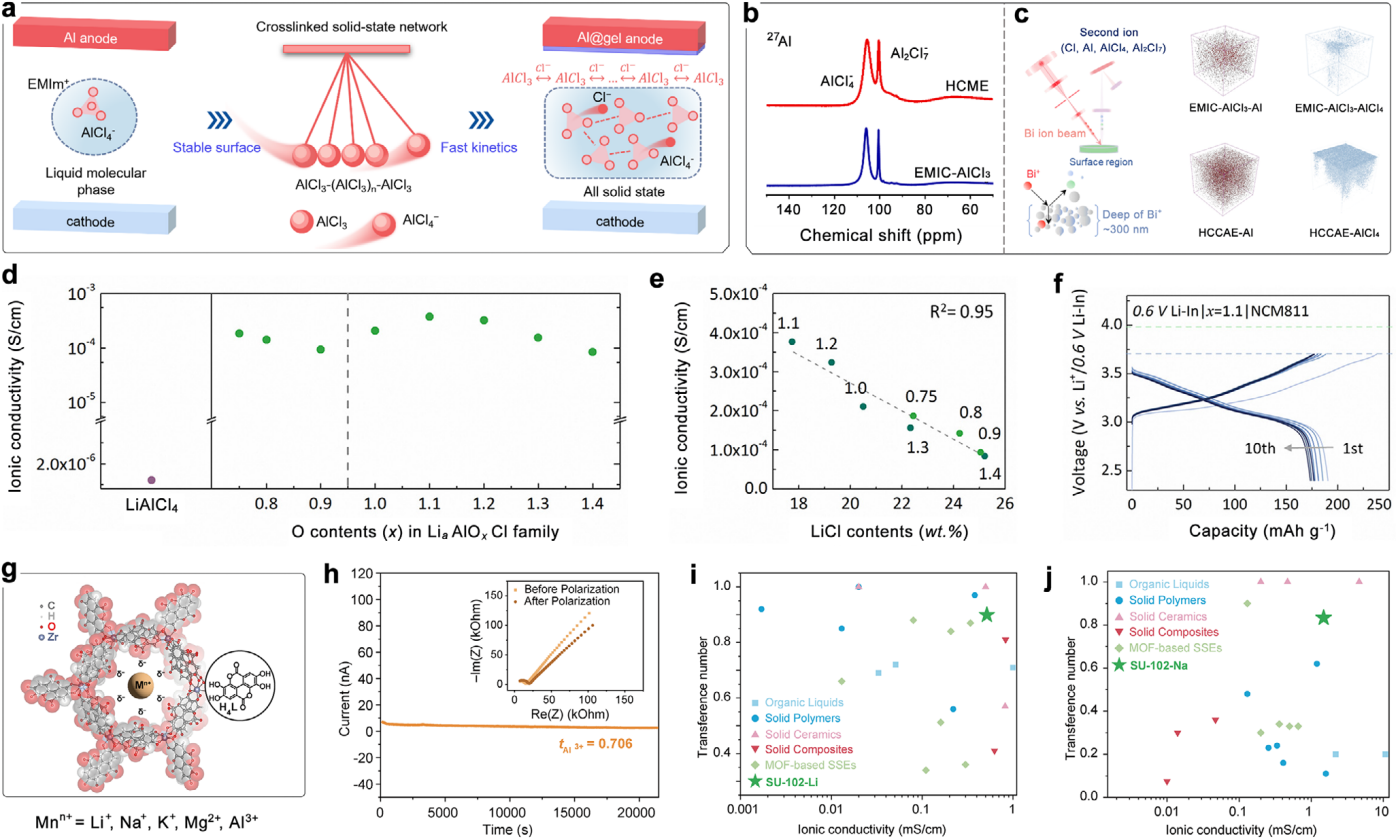
(a) Schematic evolution of electrolyte architecture: from liquid to solid‐state composite layer design. (b) ^27^Al NMR of HCCAE and ILs. (c) The sputtering schematic diagram of TOF‐SIMS, and 3D images of the sputtered volume of the graphite surface using ILs and HCCAE, corresponding to the etching time profiles. Reproduced with permission [117]. Copyright 2025, American Chemical Society. (d) Ionic conductivity of LiAlCl_4_ and the Li–Al–O–Cl family at 25 1C. (e) Relationship between the ionic conductivity of the Li–Al–O–Cl family and LiCl content. (f) Charge–discharge curves for the first ten cycles of the Li–In|x = 1.1|NCM811 full cell at 25 1C with 0.1C. Reproduced with permission [118]. Copyright 2025, The Royal Society of Chemistry. (g) Structure of SU‐102‐M viewed down the *c*‐axis. (h) Current−time profile of M|SU‐102‐M|M cells. (i,j) Performance comparison between SU‐102‐Li and SU‐102‐Na. Reproduced with permission [121]. Copyright 2026, American Chemical Society.

Addressing the challenges that conventional aluminum batteries face with chloroaluminate IL electrolytes, which rely on the dynamic equilibrium of AlCl_4_^−^ and Al_2_Cl_7_^−^ for ion conduction but suffer from narrow electrochemical windows, interfacial instability, high hygroscopicity, and susceptibility to leakage, the traditional solid‐state electrolyte design approaches are not directly applicable [64, 108]. To tackle these issues, Guo et al. [117] proposed a highly coordinated chloroaluminate electrolyte (HCCAE). By tuning the AlCl_3_/EMIC molar ratio (≥ 2:1), a chain‐like chloroaluminate network is formed: AlCl_3_−(AlCl_3_)_n_−AlCl_3_−AlCl_4_^−^ (Figure 11a). The ion transport mechanism resembles a Newton's cradle, where AlCl_4_^−^ hops between chain ends while the chains themselves remain stable, enabling continuous and rapid solid‐state conduction. The electrolyte exhibits a room‐temperature ionic conductivity of 0.89 mS cm^−1^, an electrochemical stability window > 2.6 V, and an anionic transference number of 0.46. Al||Al symmetric cells demonstrated stable cycling for over 900 h. Furthermore, the formation of the chain‐like chloroaluminate network and its role in ion transport were confirmed through DFT calculations, MD simulations, Raman spectroscopy, ^27^Al NMR, and TOF‐SIMS analyses (Figure 11b,c).

In a complementary approach, Kim et al. [118] investigated an aluminum‐based halide solid‐state electrolyte tuned through oxygen doping, aiming to address the poor reductive stability of conventional halide electrolytes and their incompatibility with low‐potential anodes. A series of amorphous Li‐Al‐O‐Cl oxychloride electrolytes was synthesized via mechanical ball milling, with oxygen content (x) and lithium sources (Li_2_O or Li_2_O_2_) varied to obtain the optimal composition [119], Li_1.1_AlO_1.1_Cl_3_ (Figure 11d). The introduction of oxygen promoted the formation of Al‐O bonds and facilitated amorphization, but also led to the precipitation of a LiCl phase. The study revealed an ionic conductivity of 3.77 × 10^−4^ S cm^−1^ at room temperature (Figure 11e) and an activation energy of 0.384 eV. When paired with a 0.6 V Li‐In alloy anode, NCM811 full cells retained 80% of their capacity (188.8 mAh g^−1^) after 250 cycles (Figure 11f). More remarkably, when matched with a 0.3 V Li‐In anode, the cell exhibited a capacity retention of 91.5% after 100 cycles, representing one of the best performances for halide electrolytes paired with low‐potential anodes. In summary, this approach significantly enhanced reductive stability while preserving high ionic conductivity [120] and achieved stable compatibility with a 0.3 V low‐potential anode through an in situ formed oxygen‐rich passivation layer. This provides a new pathway for developing low‐cost, high‐energy‐density solid‐state batteries.

Recently, Yang et al. [121] designed a quasi‐solid electrolyte based on an anionic MOF and achieved efficient transport of multiple ions, including Li+, Na^+^, and Al^3+^, through a cation exchange strategy. SU‐102, formulated as [(CH_3_)_2_NH_2_]_2_[Zr(HL)_2_] (H_4_L = ellagic acid), possesses an anionic framework with one‐dimensional (1D) channels filled with exchangeable DMA+ cations. By immersing SU‐102 in high‐concentration aqueous MClx solutions (M = Li^+^, Na^+^, K^+^, Mg^2+^, Al^3+^) (Figure 11g), near‐quantitative cation exchange was achieved, yielding the SU‐102‐M series. The materials retained their crystal structure post‐exchange, exhibiting high specific surface areas of 475–1044 m^2^/g, and large pore volumes of 0.25–0.50 cm^3^/g. Furthermore, the negatively charged SU‐102 framework contains only mobile cations (e.g., Al^3+^) within the channels [50, 71], with no free anions. This enables single‐ion conduction, effectively eliminating concentration polarization and dendrite issues caused by anion migration (Figure 11h–j). Concurrently, it facilitates a high concentration of Al^3+^ charge carriers, ultimately promoting ion transport [53].

In a word, all three approaches deviate substantially from the polymer‐ceramic composite paradigm that dominates lithium solid‐state battery research. This divergence is not accidental, the unique chemistry of aluminum, with its higher charge density, slower kinetics, and specific coordination preferences requires bespoke solutions. The field is gradually recognizing that simply translating lithium‐based designs is insufficient. Instead, new paradigms (chain‐mediated transport, oxygen‐rich passivation, anionic framework single‐ion conduction) must be developed from first principles. This represents both a challenge and an opportunity, the design space for aluminum electrolytes is much less explored than for lithium, leaving substantial room for innovation.

## 4 Performance Evaluation and Integration Challenges From Interface to Device

### 4.1 Achieving and Balancing Key Performance Indicators

Research on SSABs has achieved significant breakthroughs in key performance metrics through a series of advanced strategies in recent years [71, 78, 122]. These metrics primarily include critical current density, long‐term cycling life, and high Coulombic efficiency. However, the enhancement of these performances has not been achieved in isolation, rather, it results from a delicate balance between ionic conductivity and interfacial stability [123], high areal capacity and cycle life [124], and high‐capacity cathodes and the shuttle effect.

In terms of increasing the critical current density, core strategies involve constructing intimate interfaces through in situ polymerization and introducing ion/electron dual‐conductive interlayers [125]. Utilizing the selective adsorption of different ions by nitrogen‐doped carbon tubes [126] effectively regulates ion transport and inhibits aluminum dendrite growth, providing a stable foundation for the battery to withstand higher current densities. Second, long cycle life stands as one of the most prominent advancements in solid‐state aluminum batteries. Polymer solid‐state electrolytes fabricated via in situ polymerization not only deliver excellent room‐temperature ionic conductivity up to 4.15 × 10^−3^ S cm^−1^ [104, 105], but also, through a synergistic design with polymer‐encapsulated graphite cathodes [127], effectively constrain the volume expansion of the cathode material during cycling. This achieves remarkable stability with over 10 000 cycles and no significant capacity decay. Furthermore, addressing the issue of active material dissolution faced by high‐capacity conversion‐type cathodes (e.g., CuSe) [128], the introduction of a dual‐conductive interlayer effectively adsorbs soluble intermediates and suppresses the shuttle effect, enabling stable battery operation for over 5000 cycles at high current densities. Regarding the improvement of Coulombic efficiency, pre‐lithiation techniques precisely regulate the lithium content within the aluminum anode [129], inducing reversible phase evolution [130], and significantly reducing the irreversible capacity loss during the initial cycle.

In summary, the development of SSABs embodies the art of balancing performance metrics, the synergistic optimization between high ionic conductivity and mechanical strength, high areal capacity and volume changes, and high energy density and side reaction suppression, laying a foundation for their application in large‐scale energy storage. Additionally, a critical clarification is warranted here: the prelithiation strategy described above refers to controlled, surface‐limited prelithiation, where a thin β‐LiAl layer is formed on the Al surface via hot roll‐pressing to improve the initial Coulombic efficiency and mitigate irreversible Li loss, while the underlying Al foil remains unreacted as a structural current collector. This gradient architecture (surface β‐LiAl + bulk Al) preserves the Al‐based anode framework and should be distinguished from excessive or bulk prelithiation, where the entire Al anode is converted to Li_x_Al alloy. In the latter scenario, the active working ion becomes Li^+^, and the system would properly be classified as a LIB rather than an Al‐based battery. In our discussion, prelithiation is employed strictly as a surface activation strategy to enhance the electrochemical performance of the Al anode, rather than as a means to alter the fundamental battery chemistry. Therefore, over‐prelithiation, which would transform the battery type, is neither intended nor recommended for SSAB applications.

**FIGURE 12:**
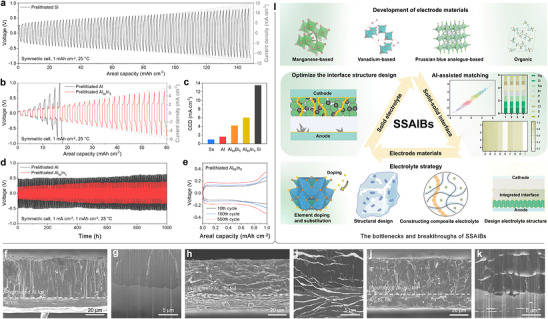
(a) CCD test of prelithiated Si electrodes in a symmetric cell with Li6PS5Cl SSE under a stack pressure of 50 MPa. (b) CCD tests of prelithiated Al and Al95In5 electrodes in symmetric cells with Li6PS5Cl SSE and a stack pressure of 50 MPa. (c) Comparison of CCD values of the prelithiated electrodes, all under a stack pressure of 50 MPa. (d) Cycling performance of prelithiated Al and Al95In5 foil electrodes in symmetric cells with 1 mA cm^−2^ current density and 50 MPa stack pressure. (e) Voltage curves from the cycling test in (d). (f–k) Cross‐sectional SEM image and Cryo‐FIB‐SEM image of a partially prelithiated Al foil electrode, a partially prelithiated Al‐In foil electrode (Al_95_In_5_), a partially prelithiated Al‐Bi foil electrode (Al_95_Bi_5_). Reproduced with permission [131]. Copyright 2025, Wiley‐VCH GmbH. (l) The bottlenecks and breakthroughts of SSABs. Reproduced with permission [134]. Copyright 2026, Elsevier.

Recently, Wang et al. [131] proposed a universal method for prelithiating alloy anodes via hot roll pressing. This process involves roll‐pressing the alloy anode with lithium foil supported on copper foil at 150°C, inducing a direct solid‐state reaction between lithium and the alloy to form a lithium‐alloy layer with a controlled thickness [74, 76]. The prelithiated electrodes exhibited initial Coulombic efficiencies exceeding 100% in half‐cells, whereas non‐prelithiated electrodes only achieved approximately 50%–70% (Figure 12a,b). In symmetric cells, the critical current density of the prelithiated Si electrode reached as high as 13.5 mA cm^−2^ (Figure 12c). The Al‐In electrode achieved 5.8 mA cm^−2^, outperforming pure Al (1.6 mA cm^−2^). In full cells paired with NMC622 cathodes, the prelithiated Si, Al, and Sn electrodes maintained good capacity retention (∼ 2.3–2.5 mAh cm^−2^) after 100 cycles, with the ICE improved to ∼ 85% (limited by the cathode) (Figure 12d,e). The multiphase Al‐In electrode could stably cycle for 400 cycles even under a low stack pressure of 2 MPa, benefiting from the surface In interfacial layer maintaining electrode/electrolyte contact. Furthermore, SEM and Cryo‐FIB‐SEM images revealed that after prelithiation (Figure 12f–k), the Al and Sn electrodes formed a bilayer structure (a surface lithium‐alloy layer atop an unreacted metal bottom), while the Si electrode formed a single Li_x_Si alloy layer [132]. In multiphase alloys (Al‐In, Al‐Bi), the higher‐potential phases (In, Bi) were preferentially lithiated, forming a 3D conductive network that facilitated rapid lithium‐ion transport [133].

Additionally, Zhang et al. [134] systematically reviewed the landscape of SSABs, spanning from fundamental scientific issues to application prospects (Figure 12l). Their analysis focused on resource cost advantages, classifications of solid‐state electrolytes, advancements in electrode materials, interfacial engineering strategies, and technology readiness assessments. The critical current density was significantly enhanced through the construction of 3D conductive networks and interfacial buffer layers [135]. Furthermore, the literature reported a critical current density (CCD) of 13.5 mA cm^−2^ for prelithiated Si anodes and 5.8 mA cm^−2^ for Al‐In alloys [136], noting that excessive prelithiation could lead to volume expansion and interfacial instability. The interfacial layer must simultaneously possess high ionic conductivity and mechanical flexibility to prevent delamination under high current densities. Moreover, the review summarized and analyzed the synergistic effect of in situ polymerized electrolytes combined with polymer‐encapsulated graphite cathodes, which enabled over 10 000 cycles without capacity decay [127]. In full cells, prelithiated anodes paired with NCM622 achieved an ICE of ∼85%, and with LCO of 91% [137, 138].

In summary, achieving a high CCD often requires sacrificing some structural redundancy [139], long‐term cycling necessitates controlling areal capacity [140], and a high ICE demands synergistic optimization of both anode and cathode [141]. Future breakthroughs lie in cross‐scale synergistic design that from tuning atomic‐level coordination environments to constructing macroscopic 3D structures, and combined with AI‐assisted material screening and intelligent manufacturing, to propel SSABs from technological validation toward engineering implementation.

### 4.2 Guiding Role of Multi‐Scale Simulation

As aluminum battery research matures, the field is increasingly moving beyond trial‐and‐error experimentation toward theory‐guided design. First‐principles calculations, molecular dynamics simulations, and high‐throughput computational screening are now being deployed to understand and predict ion transport, interfacial adsorption, and structural stability.

**FIGURE 13:**
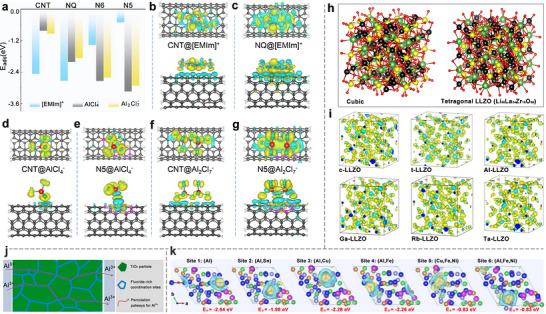
(a) Comparative adsorption energy profiles of pristine CNT and NC with distinct N configurations interacting with AlCl_4_^−^, Al_2_Cl_7_^−^, and [EMIm]^+^ species. (b) Charge redistribution patterns between pristine CNT. (c) NQ upon [EMIm]^+^ adsorption. (d) Electronic density variations of pristine CNT. (e) N5 during AlCl_4_^−^ coordination. (f) Charge density differential of pristine CNT. (g) N5 configuration interacting with Al_2_Cl_7_^−^, displayed in orthogonal projections. Reproduced with permission [47]. Copyright 2025, Wiley‐VCH GmbH. (h) Initial unit cell of cubic and tetragonal LLZO (L_i56_La_24_Zr_16_O_96_). (i) Charge density difference in c‐LLZO, t‐LLZO, Al‐LLZO, Ga‐LLZO, Rb‐LLZO, and Ta‐LLZO. Reproduced with permission [142]. Copyright 2025, MDPI. (j) Schematic representation of long‐range Al^3+^ migration through Fluorine‐rich pathways at the boundary between anatase particles. Reproduced with permission [145]. Copyright 2025, Elsevier. (k) Oxygen binding sites, with charge contours and binding energies. Reproduced with permission [146]. Copyright 2025, Wiley‐VCH GmbH.

In conventional aluminum batteries using IL electrolytes, gel polymer electrolytes (GPEs) can only partially mitigate issues such as active material dissolution and dendrite growth, but cannot completely suppress them. To address this, Li et al. [47] designed a strategy employing an ion/electron dual‐conductive interlayer (NCIL) to enhance the performance of SSABs [19]. Using first‐principles density functional theory (DFT), they calculated the adsorption energies of different nitrogen configurations (N5, N6, NQ) with ionic liquid components ([EMIm]^+^, AlCl_4_^−^, Al_2_Cl_7_^−^) (Figure 13a). NQ exhibited the strongest adsorption for [EMIm]^+,^ attributed to *π–π* interactions. N5 and N6 showed significantly enhanced adsorption for Al_2_Cl_7_^−^, originating from their electron‐withdrawing effects (p‐type doping). Furthermore, molecular dynamics simulations revealed that the NCIL promotes ion diffusion kinetics: it shortens the relaxation time for ion diffusion and reduces charge transfer resistance. Importantly, the strong adsorption of Al_2_Cl_7_^−^ by N5 and N6 sites within the NC (while exhibiting weak adsorption for Al^3+^) modulated ion migration characteristics, increasing the anion transference number from 0.15 to 0.30. This consequently promotes reaction kinetics and reduces concentration polarization (Figure 13b–g).

Recently, Raju et al. [142] primarily investigated the effects of different cation dopants (Al^3+^, Ga^3+^, Rb^+^, Ta^5+^) on the structure, electronic properties, and ion transport performance of LLZO solid‐state electrolytes. This study also employed DFT and ab initio molecular dynamics (AIMD) to systematically simulate the impact of Al, Ga, Rb, and Ta doping on the ionic conductivity, activation energy, electronic structure, defect formation energy, and binding energy of LLZO (Figure 13h). The study found that Al‐LLZO exhibited the highest ionic conductivity (1.439 × 10^−2^ S/cm) and the lowest activation energy of 0.138 eV, while also possessing an extremely low electronic conductivity (1.72 × 10^−8^ S/cm), which is beneficial for suppressing dendrite growth [143]. Furthermore, diffusion coefficients extracted from AIMD trajectories via the Nernst‐Einstein relation were used to calculate ionic conductivity and activation energy, predicting that Al doping yields the optimal performance. Additionally, charge density difference maps (Figure 13i) revealed that Al and Ta doping lead to a more uniform charge distribution, thereby lowering the electrostatic barrier for Li^+^ migration [144].

Mezzomo et al. [145] reported for the first time a class of all‐solid‐state ceramic electrolytes based on the aluminization of fluorinated TiO_2_, and systematically investigated the relationship among their structure, Al^3+^ coordination environment, and ion transport properties (Figure 13j). The migration of Al^3+^ occurs at the interfaces between anatase particles, forming hopping pathways through fluorinated surface sites. Furthermore, the diffusion trajectory of Al^3+^ between fluorinated anatase particles was simulated at the atomic scale, and the diffusion coefficient was calculated, validating the interfacial conduction mechanism.

Additionally, Anjan et al. [146] reported an aluminum‐based high‐entropy alloy (Al‐HEA) as an anode for aqueous aluminum batteries and systematically elucidated its anti‐oxidation mechanism. Through simulations, the binding energies of oxygen atoms with six different local atomic configurations on the Al‐HEA surface were calculated [147]. The pure Al site exhibited the highest binding energy of −2.64 eV (Figure 13k), although it was still lower than the −3.04 eV value for a pure Al(111) surface. The binding energy for mixed sites containing Cu, Fe, and Ni was significantly reduced (as low as −0.83 eV), indicating that these regions possess enhanced oxidation resistance. This research paradigm, encompassing “theoretical prediction, experimental validation, and mechanistic elucidation”, provides scientific guidance for the application of HEAs in aqueous aluminum batteries and opens new avenues for the design of anodes for other multivalent metal batteries [9, 10].

Collectively, these studies [47, 142, 145, 146] illustrate a profound shift in aluminum battery research from post‐hoc characterization to predictive, computation‐driven design. Li et al. [47] used DFT to predict adsorption selectivity before synthesizing the NCIL interlayer. Raju et al. [142] computationally screened multiple dopants to identify Al‐LLZO as optimal. Mezzomo et al. [145] simulated Al^3+^ diffusion trajectories to validate an interfacial hopping mechanism. Anjan et al. [146] calculated oxygen binding energies across surface configurations to explain oxidation resistance.

While these computational approaches are powerful, several caveats warrant mention. Most calculations are performed at 0 K or at simplified interfaces, potentially missing temperature‐dependent and dynamic effects. The time scales accessible by AIMD (nanoseconds) are many orders of magnitude shorter than experimental cycling times, raising questions about whether simulated diffusion pathways reflect long‐term behavior. Additionally, the field lacks standardized computational benchmarks for Al^3+^ conductors, unambiguously determining which functional and pseudopotential combinations accurately describe Al^3+^ coordination and transport remains an open challenge. Nevertheless, the integration of theory, simulation, and experiment is no longer aspirational but operational, accelerating the discovery and optimization of materials that would otherwise require years of trial‐and‐error development.

### 4.3 Challenges of Practical Device Integration

The scale‐up of SSABs from laboratory coin cells to practical pouch cells is essentially a difficult leap from a statically controllable solid–solid interface to a dynamically stable one [148]. In coin cells, the physical contact between the electrode and the electrolyte can be forcibly maintained by the megapascal‐level pressure applied by an external fixture [149]. However, in pouch cells, this substantial external pressure cannot be used in the pursuit of practical energy density, making interface management a primary challenge [150]. The volume change of the aluminum anode, which can reach up to 200% during charge and discharge, repeatedly disrupts the interfacial contact [151], leading to interrupted ion pathways and a sharp increase in internal resistance.

On an engineering level, large‐scale manufacturing faces stringent environmental control and yield challenges [152]. Sulfide solid‐state electrolytes are extremely sensitive to moisture and oxygen [153], requiring production line dew points below −60°C, which significantly increases initial investment. Furthermore, ultrathin aluminum foil is prone to wrinkling and breakage during continuous calendering, and current production yields are far lower than those for liquid electrolyte batteries [154]. Cost and sustainability also constrain industrialization; the current cost of a full solid‐state cell is approximately 2.2 RMB/Wh, which is 3−5 times that of traditional LIBs [155]. Although aluminum is an abundant resource, green recycling technologies for the entire lifecycle of the new electrolytes still need to be developed proactively. Only when systematic breakthroughs are achieved in interface engineering, manufacturing processes, and cost control can SSABs truly cross the threshold into practical application.

**FIGURE 14:**
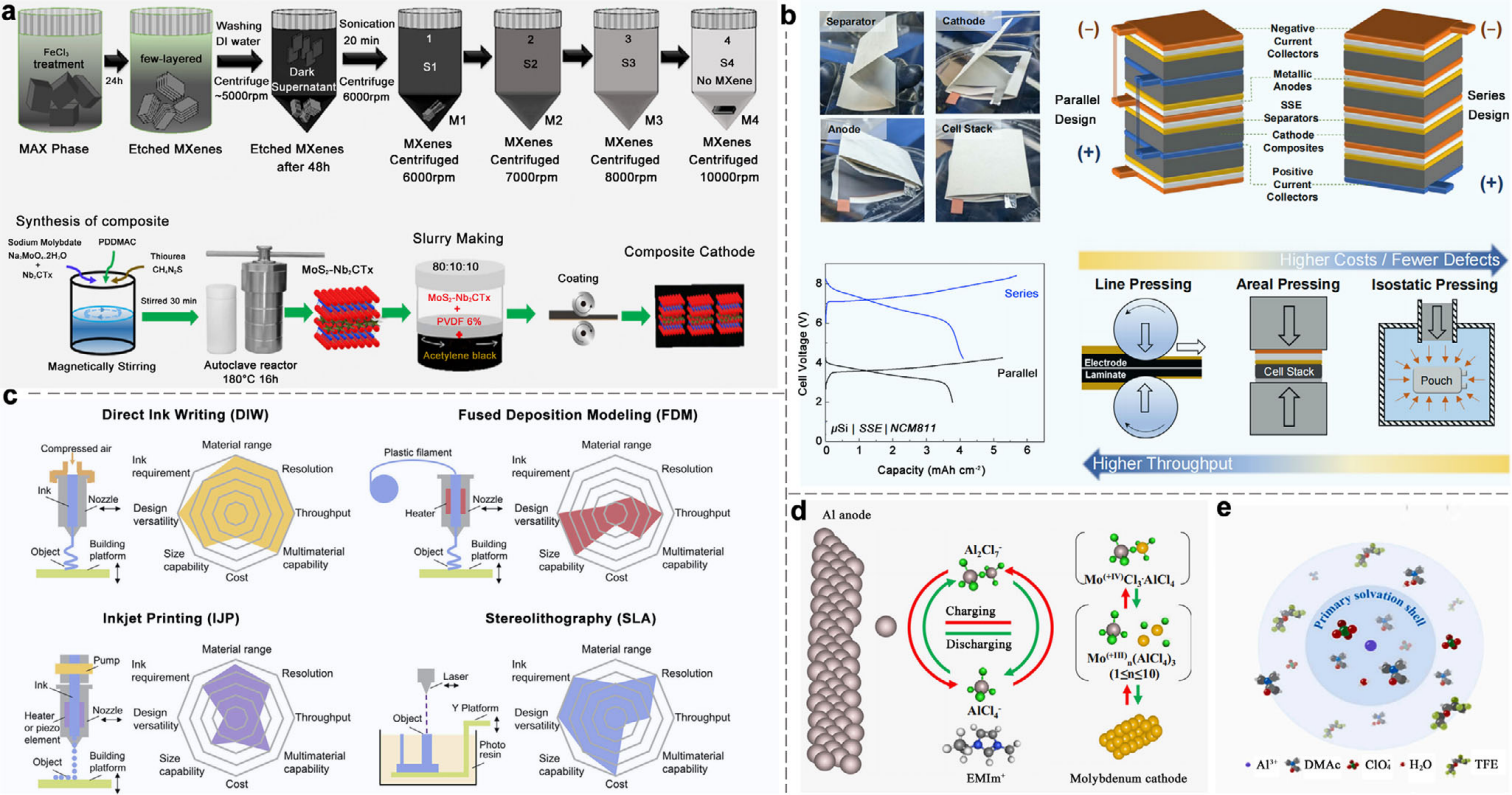
(a) Schematic representation of the synthesis of Nb_2_CT_x_ by green etchant (FeCl_3_ + TA). Synthesis of Nb_2_CT_x_‐MoS_2_ composites by the hydrothermal method. Additionally, the preparation of slurry with 80:10:10 ratio of active material, conducting carbon, and PVDF binder, respectively, followed by pasting the slurry on carbon paper is depicted. Reproduced with permission [156]. Copyright 2025, Oaepublish. (b) Cell assembly and configurations. Reproduced with permission [162]. Copyright 2022, Elsevier. (c) Schematic Principles and Features of the Main 3D Printing Techniques for Battery Manufacturing. Reproduced with permission [164]. Copyright 2020, Elsevier. (d) The proposed working mechanism of Mo‐foil in AlCl_3_‐based RAB. Reproduced with permission [165]. Copyright 2025, Wiley‐VCH GmbH. (e) Schematic diagram of the solvation structure. Reproduced with permission [166]. Copyright 2024, Elsevier.

Mahar et al. [156] reported a novel cathode material for aluminum‐ion batteries. The team synthesized Nb_2_CT_x_ MXene using a green, hydrofluoric acid‐free method and then combined it with MoS_2_ via a hydrothermal process to create a Nb_2_CTx‐MoS_2_ composite cathode (Figure 14a). This was assembled with an aluminum anode and an AlCl_3_/[BMIM]Cl ionic liquid electrolyte into a 2 × 2‐inch pouch cell. The cell delivered a specific capacity of approximately 350 mAh g^−1^ at a current density of 100 mA g^−1^ and maintained a Coulombic efficiency of 98% after 500 cycles. The research indicates that the introduction of MoS_2_ effectively suppresses the strong Coulombic interaction between the cathode and chloroaluminate ions, alleviating structural distortion during cycling and enhancing stability.

This battery uses an ionic liquid electrolyte and thus remains a liquid system, which conversely highlights the core challenge of all‐solid‐state aluminum batteries: the dynamic mismatch at the solid–solid interface [28]. In coin cells, interfacial contact can be maintained by applying megapascal‐level pressure with an external fixture, but pouch cells, designed to be thin, light, and high‐energy, cannot withstand such high external pressure. The key to engineering scale‐up lies in managing the significant volume changes of the aluminum anode and cathode during cycling under no or low external pressure [157]. The composite strategy from the paper could be adapted for electrolyte or interface design, such as developing flexible solid electrolytes or constructing adaptive buffer layers to achieve interfacial self‐healing.

Large‐scale manufacturing faces three major challenges: First, environmental sensitivity, if using sulfide solid‐state electrolytes [153], the production environment requires a dew point below −60°C, making costs far exceed those of traditional lithium batteries. Second, consistency control, achieving uniform nanoscale MoS_2_ loading on meter‐scale electrodes, is extremely difficult. Third, ultrathin aluminum foil processing [158], 0.01mm aluminum foil is prone to tearing and wrinkling during continuous calendering, making yield control a bottleneck for mass production.

Regarding cost and environment, although the synthesis step is green, the cost of ionic liquid electrolytes and MXene precursors is currently high [97, 159], and large‐scale cost reduction depends on the maturation of the industrial chain. Furthermore, the energy consumption of the hydrothermal process [160], the use of organic solvents [161], and the recycling technology for composite materials and ionic liquids from spent batteries are still underdeveloped, requiring careful assessment of the full lifecycle environmental impact. Only by overcoming these engineering challenges can SSABs truly bridge the gap from the laboratory to the market.

Tan et al. [162] systematically discussed the core challenges in scaling up high‐energy‐density sulfide‐based all‐solid‐state batteries from laboratory research to pilot production. The article points out that although solid‐state batteries hold great potential in terms of safety and energy density, their industrialization is severely hindered by the lack of scalable manufacturing processes (Figure 14b). The authors elaborately discuss the engineering issues in key steps such as solid‐state electrolyte synthesis and post‐treatment [129, 153], dry‐process electrode and separator preparation, and cell stacking and densification. They particularly emphasize the significant difference in interfacial pressure management when transitioning from coin cells to pouch cells, and the high pressure applied by external fixtures in the coin cell stage is difficult to implement in pouch cells [163], necessitating module design to maintain a uniform, low‐stress state. Furthermore, the article compares the impact of wet and dry manufacturing processes on cost, environment, and ion transport, highlighting the advantages of the dry process in reducing energy consumption and increasing electrode loading [112]. Finally, it calls for collaborative efforts among academia, national laboratories, and industry to bridge the valley of death from materials research to pilot production, thereby paving the way for solid‐state battery technology to truly enter the market.

Additionally, Lyu et al. [164] focused on the design and manufacturing techniques for 3D‐printed batteries, pointing out that this approach can construct 3D electrode structures to significantly enhance areal energy density. The study highlights four key techniques: direct ink writing (DIW), fused deposition modeling (FDM), inkjet printing (IJP), and stereolithography (SLA) (Figure 14c), and analyzes printing strategies for components such as electrodes and electrolytes [60, 61, 62].

If this concept is applied to SSABs, the core challenge for practical integration lies in leveraging the structural advantages of 3D printing to address the unique difficulties of aluminum batteries [79]. First, the substantial volume change of the aluminum anode (∼ 200%) necessitates printing porous frameworks or patterned electrolytes to provide a buffer. Second, the large size of chloroaluminate ions requires the design of electrodes with directional ion channels to shorten transport pathways [100]. Current bottlenecks include limited printing resolution, and maintaining continuity and consistency control when scaling from the laboratory to pilot production lines remains a key factor for large‐scale implementation.

Fuentes‐Mendoza et al. [165] provide a systematic review of the research challenges facing rechargeable aluminum batteries (RABs). They point out that currently, only chloroaluminate ionic liquid electrolytes can enable efficient aluminum deposition/dissolution [6, 7]. However, their strong corrosivity causes a cascade of issues, including the dissolution of cathode materials, decomposition of binders (such as PVdF), corrosion of common current collectors (stainless steel, Mo), and sensitivity to moisture and oxygen that leads to side reactions (Figure 14d). The authors emphasize the need to develop non‐corrosive electrolytes and recommend the use of PTFE binders, TiN‐coated current collectors, and corrosion‐resistant PEEK test cells. This implicitly suggests that the significant volume change of the aluminum anode is more likely to cause interfacial separation in a solid‐state system [107], necessitating the design of adaptive structures.

Furthermore, Zhao et al. [166] reported a localized high‐concentration deep eutectic electrolyte composed of hydrated aluminum perchlorate, N,N‐dimethylacetamide (DMA), and a fluorinated diluent, TFE (Figure 14e). The TFE does not participate in Al^3+^ coordination but serves to reduce viscosity, enhance ionic conductivity, and induce the formation of an AlF_3_‐rich SEI layer, significantly improving the reversibility of the aluminum anode and providing flame‐retardant safety. A full cell paired with a CuHCF cathode demonstrated excellent rate capability and cycling performance [39, 61, 62].

Considering this study, a core challenge for the practical integration of SSABs is revealed that how to transplant the interfacial regulation strategies from these liquid systems (such as fluorine‐rich SEI and localized high‐concentration structures) into an all‐solid‐state environment [21, 26, 149], while simultaneously addressing the issues of dynamic solid–solid interfacial contact and volume expansion between the solid electrolyte and both the aluminum anode and the cathode [79, 97].

The transition from coin cells to practical SSAB pouch cells requires solving a nested set of challenges across multiple length scales and disciplines. At the interface level, the 200% volume change of aluminum demands adaptive, self‐healing interfaces rather than rigid, static contacts. At the manufacturing level, environmental sensitivity, consistency control, and foil processing must be addressed through process innovation. At the cost and sustainability level, green synthesis routes and recycling infrastructure must be developed in parallel with cell technology. And at the system level, the interfacial regulation strategies proven in liquid systems must be translated to all‐solid‐state environments.

The field has made impressive progress in materials discovery and coin cell demonstration. However, the gap between these laboratory achievements and practical pouch cells remains wide. The studies reviewed here [156, 162, 164, 165, 166] collectively suggest that the most pressing need is not better materials themselves, but rather integrated approaches that simultaneously consider interfacial chemistry, mechanical design, manufacturing processability, and cost. Single‐axis optimization, pursuing ionic conductivity without regard to mechanical compliance, or energy density without regard to manufacturing yield, will continue to produce materials that work beautifully in coin cells but fail in the real world. However, the true breakthrough will come when the community treats scale‐up not as an afterthought, but as a primary design constraint from the very beginning of materials development.

### 4.4 Perspectives on Scalable Interface Engineering for Practical SSABs

While the preceding sections have systematically reviewed a wide array of interface strategies at the laboratory scale, translating these elegant designs into practical, large‐format SSABs demands a stringent evaluation of their scalability and manufacturing feasibility (Table 2). From an industrial perspective, the most practical strategies are those that not only enhance electrochemical performance but also align with existing manufacturing infrastructure, minimize additional capital expenditure (CAPEX), and maintain consistent quality over meter‐scale production. Based on these criteria, we distill the following critical perspectives:

**TABLE 2:** Assessment of typical interface strategies for SSABs regarding large‐scale manufacturing feasibility.

Interface strategy	Key advantage	Manufacturing scalability	Main bottleneck for scale‐up	Ref.
In situ polymerization	Seamless solid–solid contact, zero extra handling	High	Control of polymerization uniformity in thick electrodes	[115]
Polymer‐ceramic composites	Good processability and balanced conductivity	High	Preventing filler agglomeration over long‐term casting	[107]
ALD/CVD artificial layers	Precise, conformal coating	Very low	Extremely high cost and slow throughput	[89]
Complex 3D architectures	Large surface area, buffers volume expansion	Low	High material waste, difficult to scale	[113]
Ion/electron conductive interlayers	Suppresses the shuttle effect and dendrites	Moderate	Need for large‐area, defect‐free porous layers	[125]

Among all strategies discussed, the in situ polymerization strategy emerges as the most promising candidate for large‐scale expansion. This technique, which constructs a quasi‐solid polymer electrolyte directly within the cathode or on the electrode surface [51, 104, 105, 106], offers three distinct manufacturing advantages: (i) it circumvents the need for independent, freestanding electrolyte film handling, reducing breakage rates, (ii) it is fully compatible with existing slot‐die coating and roll‐to‐roll processes, as it relies on liquid precursor infiltration followed by thermal or UV curing, and (iii) it creates a molecular‐scale, defect‐free interface that dynamically accommodates the severe volume changes (∼ 200%) of the aluminum anode, a challenge that static coating methods fail to address.

Another highly scalable route is the composite solid electrolyte with surface‐modified ceramic fillers [92]. By employing cost‐effective surfactants (e.g., CTAB) to prevent LLZO agglomeration, this strategy enables the production of uniform, defect‐free polymer‐ceramic membranes at high speeds. Its mechanical flexibility and processability make it inherently suitable for winding processes, which are the backbone of current battery manufacturing. We note that this approach has already shown promising compatibility with high‐loading cathodes, a key step toward achieving practical energy densities.

In contrast, we must critically acknowledge the current limitations of several highly touted academic strategies. For example, the ALD/CVD deposition of artificial interfacial layers, despite offering atomic‐level precision, suffers from extremely low deposition rates and high vacuum costs, making it economically unsustainable for the > 1 GWh scale. Similarly, complex 3D‐structured anodes or electrolytes requiring photolithography or advanced templating are unlikely to be adopted in the near term due to low throughput and high material waste.

Furthermore, the multifunctional ion/electron dual‐conductive interlayers (e.g., N‐doped carbon layers [47]) represent a double‐edged sword. While they effectively adsorb soluble intermediates and suppress dendrites, their practical implementation requires precise, large‐area coating of porous carbon layers with uniform thickness and porosity. Current laboratory methods (e.g., vacuum filtration or manual coating) are not directly transferable to continuous manufacturing, and developing scalable, high‐speed coating slurries remains an open challenge.

In summary, we propose that future industrial efforts should prioritize process‐compatible strategies, particularly in situ polymerization and polymer‐ceramic composite engineering, as they offer the most balanced trade‐off between performance enhancement and manufacturing simplicity. Concurrently, the community should actively pursue process innovation for other promising strategies, for instance, developing roll‐to‐roll compatible ALD systems or water‐based, high‐solid‐content slurries for interlayer coatings. Ultimately, the transition of SSABs from coin cells to pouch cells will depend not on a single hero strategy, but on an integrated approach where interface chemistry is designed a priori with manufacturing constraints in mind.

## 5 Conclusion and Prospect

The current scientific consensus on the interface challenges of SSABs centers on the dynamic mismatch of the solid–solid interface [167]. The significant volume change of the aluminum anode (∼ 200%) during cycling, coupled with the intercalation and deintercalation of bulky chloroaluminate complex ions [168, 169], leads to repeated contact loss between electrode and electrolyte, thereby disrupting ion transport pathways [97]. Unlike lithium systems, where volume changes are comparatively modest, aluminum's extreme expansion creates a moving target for interfacial stability, a problem that static interface designs cannot solve.

The most effective engineering solutions exhibit a dual‐track strategy of liquid‐assisted + solid‐stabilized approaches [170]. On one hand, leveraging interface regulation strategies from liquid electrolyte systems [171], such as using fluorine‐containing diluents to induce formation of an AlF_3_‐rich SEI layer or employing localized high‐concentration concepts to stabilize the solvation structure [172], provides proven chemical solutions. On the other hand, constructing adaptive interfacial layers in the solid‐state environment, including flexible polymer buffer layers [173], patterned electrolyte surfaces to increase contact area [174], and 3D porous frameworks to accommodate volume expansion [175], addresses the mechanical aspects of the problem. Critically, the dry electrode process [176], due to its low energy consumption, high loading capacity, and excellent interfacial integration, is regarded as the most promising scalable fabrication route. This dual‐track approach acknowledges a fundamental truth, no single strategy can simultaneously address the chemical, mechanical, and dynamic aspects of the SSAB interface.

**FIGURE 15:**
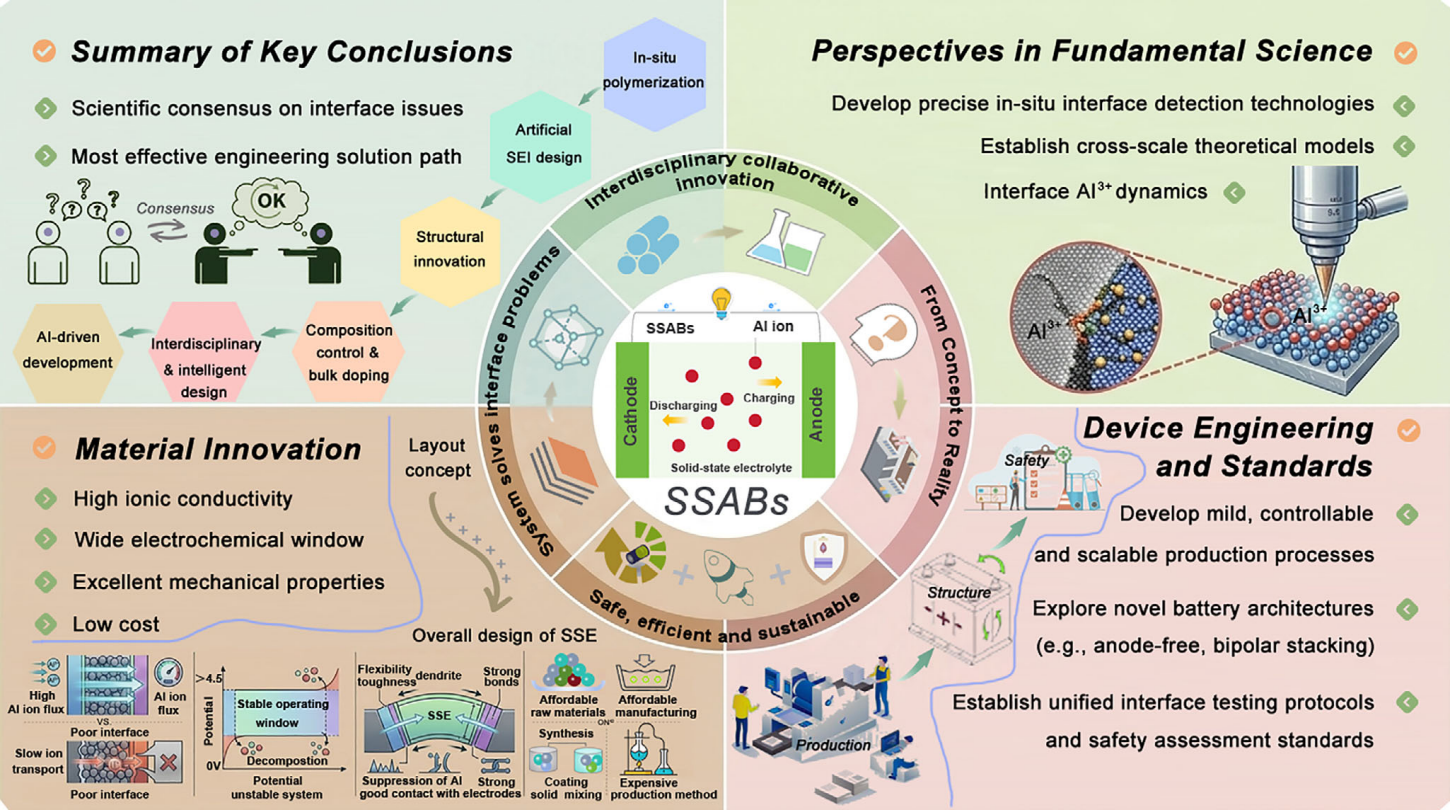
Conclusion and the prospect of SSABs.

Based on the concept of overcoming interfacial barriers in SSABs for safe and high‐energy‐density storage, we systematically summarize the physicochemical origins of multi‐scale interfacial challenges in these systems, focusing on the latest breakthrough strategies in interface design and engineering. The discussion delves into how constructing artificial interlayers, designing gradient composite electrolytes, regulating interfacial ion transport channels, and introducing novel interfacial characterization techniques can effectively promote uniform Al^3+^ deposition and stripping, suppress side reactions, and achieve stable interfacial contact. This integrated approach paves viable paths toward achieving high ionic conductivity, excellent mechanical strength, and electrochemical stability simultaneously, ultimately providing a clear roadmap (Figure 15) for the development of next‐generation SSABs capable of both high energy density and long‐term safe operation.

Therefore, future research directions will mainly focus on the following four aspects:

1. At the fundamental science level, there is an urgent need to develop in situ/operando interfacial probing techniques (e.g., synchrotron XRD, ambient‐pressure XPS, cryo‐EM) to track Al^3+^ migration and reactions at the interface in real‐time [177, 178, 179]. Establishing multi‐scale theoretical models that integrate first‐principles calculations with phase‐field simulations is crucial for predicting interface evolution [180]. Furthermore, an in‐depth understanding of the solvation/desolvation behavior of Al^3+^ at complex interfaces is required to elucidate the energy barriers and kinetics of multivalent ions crossing the solid–solid interface.
2. Regarding materials innovation, the design of ideal interfacial layers and solid‐state electrolytes must simultaneously meet requirements for high ionic conductivity (> 1 mS/cm), wide electrochemical window (> 4 V), excellent mechanical flexibility, and low cost [181, 182, 183, 184]. Promising directions include biomimetic gradient interfaces [185], MOF/COF‐based ion sieves [186], and composite solid electrolytes (e.g., sulfide‐polymer hybrids) [187].
3. For device engineering, it is essential to develop mild, controllable, and scalable interface construction processes, such as in situ polymerization [188], vapor deposition [189], and 3D‐printed structured electrodes [190]. Exploring anode‐free configurations to circumvent aluminum foil pretreatment issues and bipolar stacking to enhance cell voltage and energy density are also critical.
4. In terms of standards and evaluation, there is a call to establish unified interface performance testing protocols (e.g., critical current density, standard interface impedance evolution) and safety assessment standards (e.g., nail penetration, overcharge, thermal runaway thresholds) to provide a basis for technology comparison and industrialization access.

In summary, overcoming the interfacial barriers in SSABs requires coordinated progress across four interconnected fronts for fundamental science (to understand the problem), materials innovation (to solve the chemistry), device engineering (to scale the solution), and standards (to compare and validate). The dual‐track liquid‐assisted + solid‐stabilized strategy recognizes that hybrid approaches borrowing from both liquid and solid electrolyte paradigms may be more immediately fruitful than purely solid‐state solutions. The most promising path forward lies not in any single breakthrough but in the systematic integration of advanced characterization, rational materials design, scalable manufacturing, and standardized evaluation. Only then can SSABs transition from scientific curiosities to practical, high‐energy‐density, safe energy storage systems.

## Author Contributions

**Yunlei Wang**: conceptualization, methodology, funding acquisition, writing – original draft, writing – review and editing. **Xiaogang Zhang**: conceptualization, validation, data curation, resources, writing – review and editing. **Taibin Wu**: writing – review and editing, conceptualization, supervision, resources. **Mingguang Wang**: writing – review and editing, conceptualization, investigation, resources. **Shitao Dou**: conceptualization, writing – review and editing, resources, data curation. **Yifan Wu**: conceptualization, methodology, data curation, resources.

## Conflicts of Interest

The authors declare no conflicts of interest.

## Data Availability

Research data are not shared.
